# Landscape of transcription and long non-coding RNAs reveals new insights into the inflammatory and fibrotic response following ventilator-induced lung injury

**DOI:** 10.1186/s12931-018-0822-z

**Published:** 2018-06-22

**Authors:** Lu Wang, Nannan Zhang, Yi Zhang, Jingen Xia, Qingyuan Zhan, Chen Wang

**Affiliations:** 1 Beijing University of Chinese Medicine, No 11, East Bei San Huan Road, Chaoyang District, Beijing, 100029 China; 2 Center for Respiratory Diseases, China-Japan Friendship Hospital, No 2, East Yinghua Road, Chaoyang District, Beijing, 100029 China; 3 Department of Pulmonary and Critical Care Medicine, China-Japan Friendship Hospital, No 2, East Yinghua Road, Chaoyang District, Beijing, 100029 China; 4 National Clinical Research Center for Respiratory Diseases, No 2, East Yinghua Road, Chaoyang District, Beijing, 100029 China; 5 Chinese Academy of Medical Sciences and Peking Union Medical Collage, No 9, Dong Dan San Tiao, Dongcheng District, Beijing, 100730 China

**Keywords:** RNA-seq, Ventilator-induced lung injury, Lung fibrosis, LncRNAs

## Abstract

**Background:**

Mechanical ventilation can cause ventilator-induced lung injury (VILI) and lung fibrosis; however, the underlying mechanisms are still not fully understood. RNA sequencing is a powerful means for detecting vitally important protein-coding transcripts and long non-coding RNAs (lncRNAs) on a genome-wide scale, which may be helpful for reducing this knowledge gap.

**Methods:**

Ninety C57BL/6 mice were subjected to either high tidal volume ventilation or sham operation, and then mice with ventilation were randomly allocated to periods of recovery for 0, 1, 3, 5, 7, 14, 21, or 28 days. Lung histopathology, wet-to-dry weight ratio, hydroxyproline concentration, and transforming growth factor beta 1 (TGF-β1) levels were determined to evaluate the progression of inflammation and fibrosis. To compare sham-operated lungs, and 0- and 7-day post-ventilated lungs, RNA sequencing was used to elucidate the expression patterns, biological processes, and functional pathways involved in inflammation and fibrosis.

**Results:**

A well-defined fibrotic response was most pronounced on day 7 post-ventilation. Pairwise comparisons among the sham and VILI groups showed a total of 1297 differentially expressed transcripts (DETs). Gene Ontology analysis determined that the stimulus response and immune response were the most important factors involved in inflammation and fibrosis, respectively. Kyoto Encyclopedia of Genes and Genomes analysis revealed that mechanistic target of rapamycin (mTOR), Janus kinase-signal transducer and activator of transcription (JAK/STAT), and cyclic adenosine monophosphate (cAMP) signaling were implicated in early inflammation; whereas TGF-β, hypoxia inducible factor-1 (HIF-1), Toll-like receptor (TLR), and kappa-light-chain-enhancer of activated B cells (NF-κB) signaling pathways were significantly involved in subsequent fibrosis. Additionally, 332 DE lncRNAs were identified and enriched in the processes of cellular and biological regulation. These lncRNAs may potentially regulate fibrosis through signaling pathways such as wingless/integrase-1 (Wnt), HIF-1, and TLR.

**Conclusions:**

This is the first transcriptome study to reveal all of the transcript expression patterns and critical pathways involved in the VILI fibrotic process based on the early inflammatory state, and to show the important DE lncRNAs regulated in inflammation and fibrosis. Together, the results of this study provide novel perspectives into the potential molecular mechanisms underlying VILI and subsequent fibrosis.

**Electronic supplementary material:**

The online version of this article (10.1186/s12931-018-0822-z) contains supplementary material, which is available to authorized users.

## Background

During the past several decades, mechanical ventilation (MV) has played an essential role in the clinical management of patients with acute respiratory distress syndrome (ARDS). However, numerous studies have demonstrated that MV is imperfect and can cause ventilator-induced lung injury (VILI) [1, 2], a common condition that pathologically manifests as an influx of neutrophils, release of inflammatory cytokines, increased alveolar exudation, and non-cardiogenic pulmonary edema [3].

Several major mechanisms of VILI have been described including barotrauma, volutrauma, atelectrauma, and biotrauma. Barotrauna and volutrauma are caused by alveolar overdistension, and atelectrauma is due to the cyclic collapse/reopening of lung units. Biotrauma is considered to be amplification of the pro-inflammatory cascade based on a pre-existing lung injury [3, 4]. The translocation of mediators and pathogens from the alveolar spaces into systemic circulation may result in increased alveolar–capillary permeability, pulmonary edema, or even fatal multiple organ dysfunction and death [4].

Recognition of the importance of VILI has led to a marked conversion on the philosophy underlying the provision of MV. A series of randomized controlled trials were performed to determine feasible ventilation strategies that minimize lung injury. Recently, a lung-protective strategy was validated to reduce VILI following clinical recommendations based on the ARDS Network study [1, 5–7]. This investigation was a landmark study in ventilation development, and underscored the fact that a low tidal volume (V_T_) strategy with appropriate positive end-expiratory pressure (PEEP) is necessary to prevent excessive lung stretching during adjustments to MV [7].

Despite available advances in ventilation strategies, many patients eventually die after surviving the acute phase, often with evidence of pulmonary fibrosis. A prospective cohort study by Martin et al. [8] reported that in the 64% of ARDS patients diagnosed with pulmonary fibrosis there was a 57% fatality rate, while there were no fatalities in patients without fibrosis. Subsequently, other clinical trials were performed, which showed that ARDS patients with increased levels of transforming growth factor beta 1 (TGF-β1) and procollagen type III had extremely high mortality rates [9–11] than those with lower levels. Consistent with these clinical studies, basic research studies have demonstrated that MV, either of high V_T_ or high peak airway pressure, can induce lung fibrosis as early as 1 week after injury [12–14]. Therefore, MV is being increasingly recognized as a pivotal factor in the initiation or propagation of ARDS-associated lung fibrosis regardless of the original disease [8, 9, 15]. Biotrauma is considered to be the major mechanism that sets the stage for the development of subsequent fibrosis. The extracellular and intercellular mediators, either directly released by injured cells or indirectly activated by pulmonary epithelial, endothelial, or immune cells through various cell-signaling pathways, are the key biological forces that drive continuation of the fibroproliferative response [3, 9]. Recently, Lv et al. [16] proposed that the endothelial-mesenchymal transition also contributes to abnormal modulation following mechanical injury; however, these observations are still limited in scope.

Global transcriptome analysis is an emerging, powerful tool used to reveal variability in pathophysiology on a genome-wide scale [17]. This type of analysis is potentially a better way to address the knowledge gap of mechanism for VILI and subsequent fibrosis. In this study, we established a well-defined and standard “one-hit” mouse model of lung fibrosis, and then delineated a complete transcriptome image identifying all involved differentially expressed transcripts (DETs) as well as long non-coding RNAs (lncRNAs) using global transcriptome analysis. To the best of our knowledge, this is the first transcriptome study to reveal a broad spectrum of dysregulated transcripts and potential molecular pathways involved in fibrosis following VILI, as well as the first study to focus on dysregulated or dysfunctional lncRNAs that may be responsible for the pathogenesis of early inflammation or subsequent fibrosis. Together, the results of this study provide novel insights into the inflammatory and fibrotic responses following VILI.

## Methods

### Animal protocols

Male, 8–12-week-old C57/BL6 mice were randomized to the high V_T_ MV group (*n* = 80) or sham-operated group (sham, *n* = 10). After anesthetizing mice with an intraperitoneal injection of pentobarbitone (100 mg/kg; Pfizer, Dublin, Ireland), mice were intubated using a 22 G Teflon catheter and continuously ventilated for 4 h in a volume-controlled mode with a small animal ventilator (55–7040, VentElite; Harvard Apparatus, Holliston, MA, USA). The protocol comprised the following settings: V_T_ of 20 mL/kg, PEEP of 0 cm H_2_O, respiratory rate of 80 breaths/min, inspiratory-expiratory ratio of 1:1, and fraction of inspired O_2_ of 0.4 [18]. During the experimental period, mice were given an anaesthetic as needed; cocuronium besylate (0.6 mg/kg, H20130486; MSD Performance Products, Kenilworth, NJ, USA) was added for muscle relaxation. Anesthetized, intubated, non-ventilated animals served as the sham controls. The study was approved by the Institutional Animal Care and Use Committee of Capital Medical University (No. AEEI-2016-168; Beijing, China) and strictly conducted according to the University’s guidelines.

### Specimen collection and processing

Mice were sacrificed by anesthesia overdose at 0, 1, 3, 5, 7, 14, 21, and 28 days. The left lungs were weighed, dried in an oven (60 °C for 72 h), and weighed again to determine the lung wet-to-dry weight ratio [19]. The right upper lobes were used for hydroxyproline concentration measurement using a hydroxyproline assay kit (A030–2; Nanjing Jiancheng Bioengineering Institute, Nanjing, China), while the middle and dorsal segments were analyzed by quantitative PCR (qPCR) and RNA sequencing, respectively.

### Bronchoalveolar lavage fluid preparation

Preparation of bronchoalveolar lavage fluid (BALF) consisted of three separate lavages via a tracheal catheter with 3 mL sterile, ice-cold phosphate-buffered saline (PBS). For each lavage, 0.7–0.8 mL fluids were recovered and separately centrifuged at 1500 rpm for 5 min at 4 °C. Cell-free supernatants were separated for assessment of TGF-β1 using an enzyme-linked immunosorbent assay (ELISA) kit (MB100B; R&D Systems Inc., Minneapolis, MN, USA).

### Lung histopathology and immunohistochemistry

After infusion with PBS, lung samples were fixed in 10% neutral formalin for 1 week, and then embedded and sectioned for histological evaluation by hematoxylin and eosin (H&E) staining, Masson’s trichrome, and Sirius technique. Collagen deposition areas and type alterations were calculated to provide more compelling data on fibrosis. The relative expression of alpha smooth muscle actin (α-SMA) was subsequently evaluated by immunohistochemical staining with specific primary antibodies (1:100, A2547; Sigma-Aldrich, St. Louis, MO, USA). The mean density of the positive areas in the sections (at least five random microscopic fields per lung section) was calculated using Image Pro-Plus 6.0 software (Media Cybernetics Inc., Rockville, MD, USA).

### Preparation for sequencing

RNA was isolated using TRIzol reagent (15,596,026; Invitrogen, Carlsbad, CA, USA), and the RNA purity and integrity were assessed using the NanoPhotometer spectrophotometer (Implen Inc., Westlake Village, CA, USA) and the Bioanalyzer 2100 RNA Nano 6000 Kit (Agilent Technologies, Savage, MD, USA), respectively. A total amount of 3 μg RNA per sample was used as the input material. After the removal of ribosomal RNA (Epicentre Ribo-Zero TM rRNA Removal Kit; Epicentre, Madison, WI, USA), sequencing libraries were generated from the rRNA-depleted RNA using the NEBNext® Ultra™ Directional RNA Library Prep Kit for Illumina® (New England Biolabs, Ipswich, MA, USA) according to the manufacturer’s instructions. PCR was performed with Phusion High-Fidelity DNA polymerase, Universal PCR primers, and the Index (X) Primer. After clustering of the purified index-coded sampler (TruSeq PE Cluster Kit v3-cBot-HS; Illumina, San Diego, CA, USA), the libraries were sequenced on the Illumina HiSeq 2500 platform, and 125 bp (base pair) paired-end reads were produced.

### Read alignment and transcript assembly

Quality control of raw reads was performed with FastQC software (v.0.11.5). After clipping Illumina adapter sequences and trimming low-quality bases through in-house Perl scripts, high-quality clean reads were mapped against the reference genome (Ensembl, release v.87) using Tophat 2.0.9 [20] with the default parameter settings. Then the resulting aligned reads were subjected to Scripture (beta2) and Cufflinks 2.1.1 to assemble the aligned reads into genes [21].

### Identification of lncRNAs

Cuffmerge was used to merge the assembled transcripts, and those with low expression and shorter than 200 bp were abandoned. The remaining transcripts predicted with coding potential through the CNCI [22], CPC [23], Pfam-scan [24], and phyloCSF [25] tools were also filtered out, and those without coding potential were identified as the candidate set of lncRNAs.

### Quantification and analysis of differentially expressed genes

Cufflinks was used to measure the relative abundance of each transcript by calculating the fragments per kilo-base of exon per million fragments mapped (FPKM) of mRNA (coding gene) in each sample. Differential transcript expression between each pair of samples was analyzed using Cuffdiff (v 2.1.1). The calculated *P* values were subjected to the Benjamini-Hochberg method to control for a false discovery rate (FDR) [26]. Statistical significance was defined as a *P* value < 0.05 and estimated absolute Log2-fold change > 1.

### Target gene prediction of lncRNAs

Accordingly, we predicted the target genes for lncRNAs using co-location (*Cis*) and co-expression (*Trans*) analysis [27]. The co-localization threshold was set as 100 kb upstream and downstream of each lncRNA, and lncRNA targets were identified by the expressed correlation between lncRNA and coding genes, with an absolute value of correlation greater than 0.95.

### Functional enrichment analysis

KO-Based Annotation System (KOBAS) 3.0 (http://kobas.cbi.pku.edu.cn/) is a web server for functional annotation and functional set enrichment of genes [28]. For the DETs and predicted lncRNA targets, Gene Ontology (GO) and Kyoko Encyclopedia of Genes and Genomes (KEGG) pathway enrichment analysis was performed on KOBAS 3.0 [29]. Statistical significance was assessed using the hypergeometric test or Fisher’s exact test with the FDR correction method (Benjamini and Hochberg), and the threshold was set as corrected *P* values less than 0.05.

### Regulatory network of lncRNAs and mRNAs

An lncRNA–mRNA network was built to identify the interactions between mRNA and lncRNA. The networks were built according to the target relationship between mRNA and lncRNA, as identified by co-location and co-expression analyses. These differentially expressed mRNAs and lncRNAs were retained for network construction and visualized by Cytoscape 3.3 [30]

### Quantitative PCR

Total RNA from the lung tissue was extracted using TRIzol reagent (15,596,026; Invitrogen) and cDNA was generated using the High-Capacity cDNA Reverse Transcription Kit (4,368,814; Invitrogen). The PCR reaction was executed in the iQ5 system (Bio-Rad, Hercules, CA, USA) for 44 cycles, with each cycle consisting of denaturation at 95 °C for 45 s, annealing at 60 °C for 60 s, and an extension at 72 °C for 1 min using Applied Biosystems® Power SYBR® Green (4,367,659; Invitrogen). Primers are shown in the online supplement (Additional file 1: Figure S1). The abundance of each gene was normalized to that of 18S mRNA, and the fold changes were calculated with the 2^-ΔΔ^CT method [31].

### Statistical analysis

All of the data were analyzed and presented using GraphPad Prism 7.0 (Prism software; GraphPad, San Diego, CA, USA). Comparisons between two groups were determined by the Student’s *t*-test or Mann–Whitney test, and among multiple groups using one-way analysis of variance (ANOVA). *P* < 0.05 was considered statistically significant.

## Results

### Identification of the animal model

**Fig. 1:**
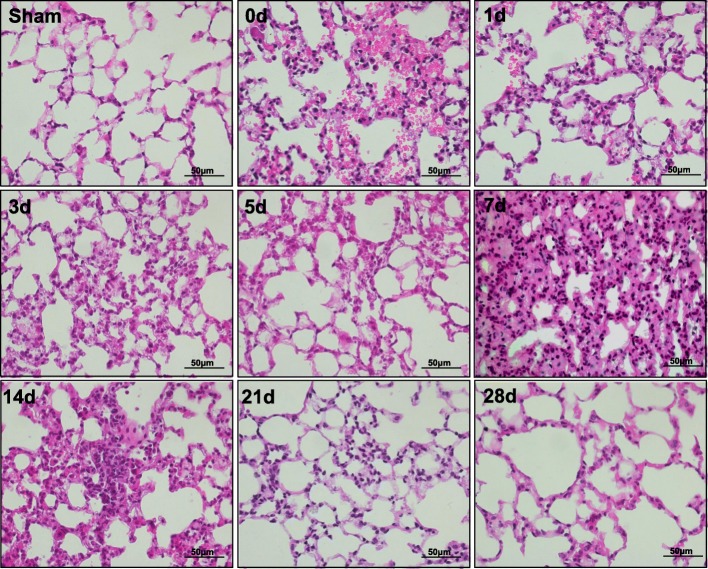
Time course of histologic injury and resolution following ventilation. Representative histological image of lung sections stained with H&E. VILI induced early inflammatory injury with significant alveolar hemorrhage and inflammatory infiltration, which was followed by a marked fibrotic response on day 7 after ventilation (original images, 400× magnification; scale bar = 50 μm)

**Fig. 2:**
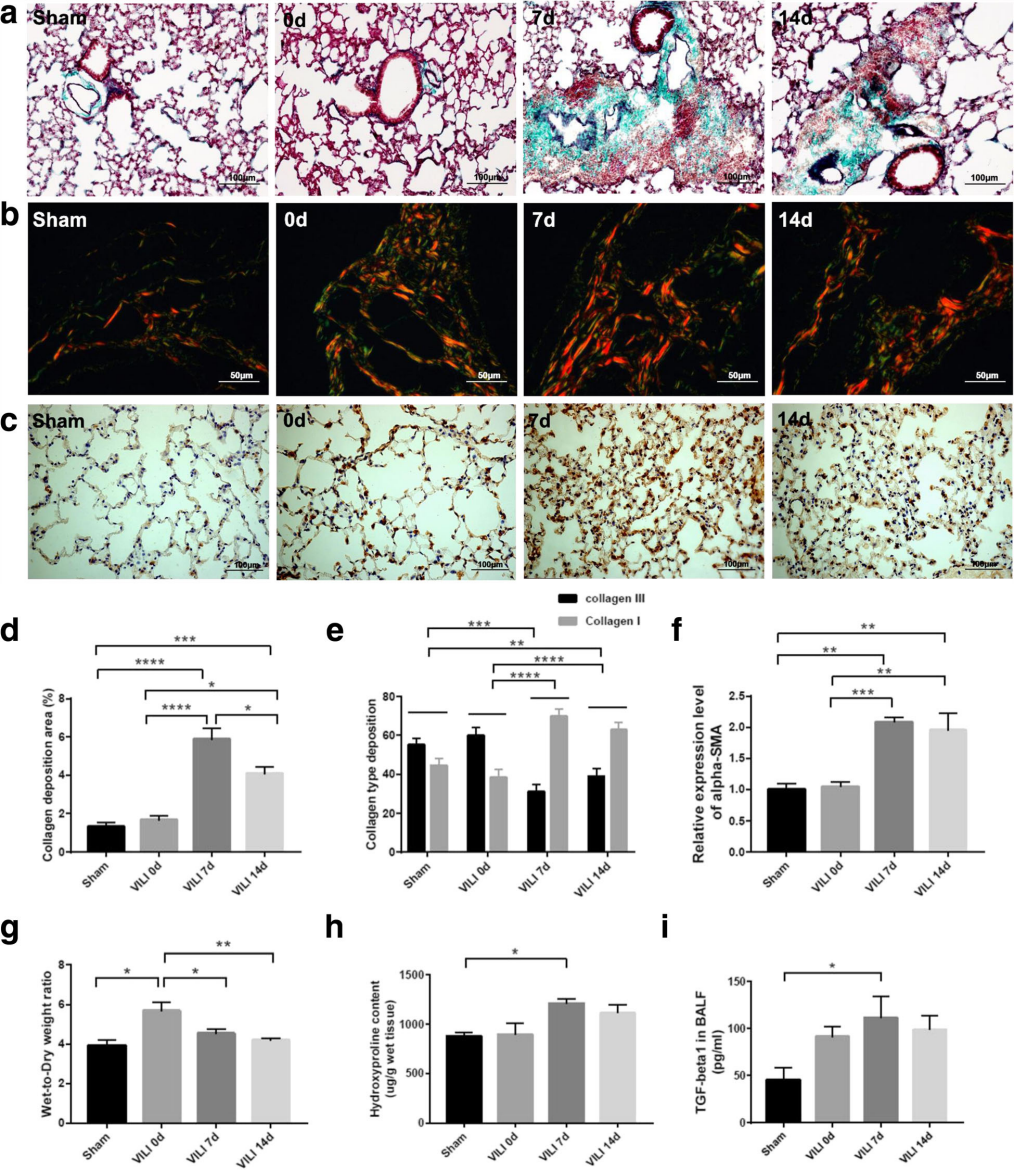
Identification of animal model**. a** Masson staining. **b** Sirius Red staining, red and green color indicated collagen I and collagen III, respectively. **c** α-SMA staining. **d** The comparisons of collagen deposition area. **e** The comparisons of lung collagen type. **f** The relative expression level of α-SMA in groups. **g** Lung wet-to-dry weight ratio. **h** Lung hydroxyproline content. **i** The expression level of TGF-β1 in bronchoalveolar lavage fluid (BALF). Statistical analysis was carried out with one-way ANOVA, and the significance level was set at: *P < 0.05, **P < 0.01, ***P < 0.001 and ****P < 0.0001

To determine the time course of repair, mice were sacrificed at predefined time points. H&E staining was used to assess the time course of histologic injury and resolution post-ventilation (Fig. 1). Masson trichrome (Fig. 2a), the Sirius technique (Fig. 2b), and α-SMA staining (Fig. 2c) were performed, and the corresponding collagen deposition areas (Fig. 2d), collagen type deposition (Fig. 2e), and positive expression of α-SMA (Fig. 2f) were determined. As shown in Fig. 1, mice with sustained ventilation had extensive alveolar damage and interstitial edema, hemorrhage, and inflammatory cellular infiltration, which gradually resolved after 24 h. After 3 days, tissue sections showed a decline in pulmonary inflammatory response, and by the 5th day, only a subtle architecture disorder remained. On day 7, significant lesions developed, mainly characterized by a thickened alveolar septum, cell proliferation, and considerable disruption of the alveolar architecture. One week later, the pathological disorders were partially attenuated, and the lungs were almost restored to normal at 1 month. Masson staining showed progressively increased collagen deposition 7 days after mechanical stress, which formed a signature morphologic lesion of pulmonary fibrosis. Intriguingly, the alterations of collagen type depicted by Sirius red were similar to those seen in bleomycin-induced pulmonary fibrosis. These results showed that type III collagen (green) was mainly expressed in lungs with early impairment, which decreased while type I collagen (red) gradually increased. Additionally, the wet-to-dry weight ratio peaked on day 0 and returned to baseline within 7 days (Fig. 2g). The lung hydroxyproline content (Fig. 2h) and level of TGF-β1 in BALF (Fig. 2i) were both elevated compared to levels in the sham group.

### Transcript analysis in lungs

**Fig. 3:**
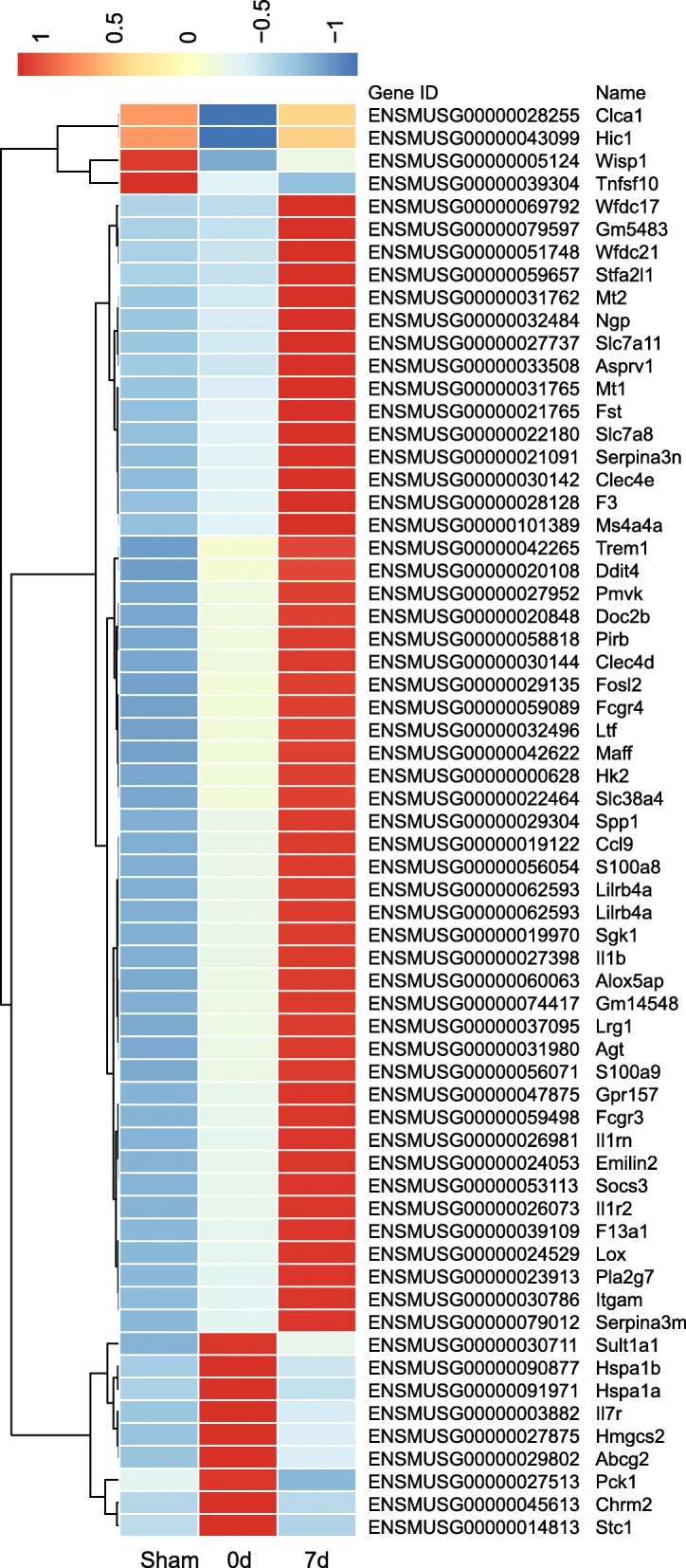
Differentially expressed transcripts (DETs) in lungs. Heat map for hierarchical clustering of all of the involved DETs in the comparisons of each pair. In clustering analysis, upregulated and downregulated genes are colored in red and blue. The heat map was performed based on the normalized fragments per kilo-base of exon per million fragments mapped (FPKM) of each DET. Differential expression analysis used a model based on the negative binomial distribution, and transcripts with *p* < 0.05 and fold change > 2 were regarded as DETs

To identify the transcriptomic gene profile, six ventilated mice (time intervals: day 0 and 7 days post-ventilation), and three sham-operated mice were utilized. The entire list of DETs was uploaded as supplementary material (Additional file 2: Table S1), and the numbers of dysregulated mRNAs were further profiled using the Volcano plot and Venn diagram (Additional file 3: Figure S2). In total, 1297 DETs were identified as having either upregulated or downregulated expression levels. In comparing the sham and VILI groups on day 0, there were 264 upregulated and 65 downregulated transcripts, whereas a total of 322 upregulated and 164 downregulated DETs were identified on day 7 post-ventilation. Furthermore, 742 upregulated and 391 downregulated transcripts were found when the VILI and sham groups were compared on day 7. In the same comparison, we also identified 63 commonly shared genes that participated in the disease (Fig. 3). Annotation was made via the reference genome Ensembl, based on the biomedical literature and integrated databases, which allowed the description of related functional and biochemical functions in which the transcripts of interest were involved. More specially, 79 genes were profiled that encoded transcription factors (TFs) with critical regulatory roles in cell activities, organ development, repair, or processes related to homeostasis and human disease, most of which have not been previously associated with VILI or lung fibrosis (Additional file 4: Table S2).

### Clustering of biological processes and functional pathway analysis

**Fig. 4:**
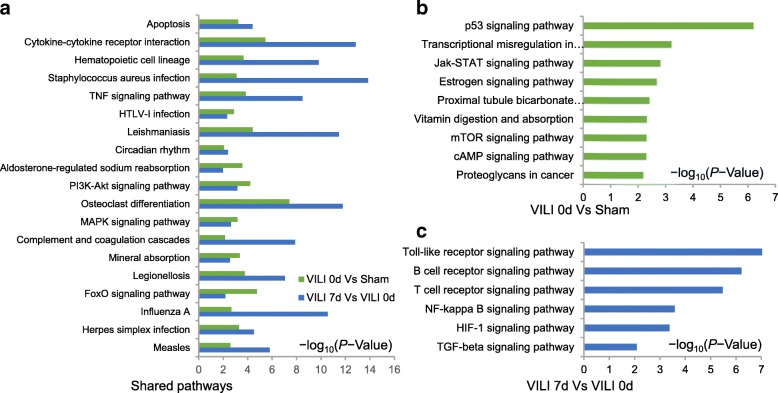
Pathway analysis. **a** Histogram showing the pivotal enriched pathways in both stages of inflammation and fibrosis. **b** Histogram indicated the significant pathways when the VILI and sham groups were compared on day 0. **c** Histogram indicated the significant pathways in the comparison between day 7 and day 0 in the VILI group. All analyses were performed using KOBAS 3.0 and statistical significance was assessed using the hypergeometric test or Fisher’s exact test with the false discovery rate (FDR) correction method

GO analysis revealed that the response tostimulus, cellular response to stimulus, response to stress and responses to chemical and cell communication were the most prominent biological behaviors and molecular functions involved in the early phase of lung injury. The categories of immune response, response to stimulus, defense response, immune system processes, and response to stress appeared to play crucial roles in formation of the fibrotic signature (Additional file 5: Figure S3, Additional file 6: Table S3). KEGG analysis was implemented to determine the most probable functional pathways associated with all of the DETs. As shown in Fig. 4, tumor necrosis factor (TNF), phosphoinositide 3-kinase/Akt (PI3K/Akt), mitogen-activated protein kinase (MAPK), and forkhead box protein O (FOXO) signaling pathways were regulated during the lung pathophysiology after ventilation. Combined with the prior literature, we speculated that the mechanistic target of rapamycin (mTOR), Janus kinase-signal transducer and activator of transcription (JAK/STAT), and cyclic adenosine monophosphate (cAMP) signaling pathways were more significant in early inflammation, whereas TGF-β, hypoxia inducible factor-1 (HIF-1), Toll-like receptor (TLR), factor kappa-light-chain-enhancer of activated B cells (NF-κB), as well as T-cell receptor and B-cell receptor signaling pathways played more prominent roles during the progression of fibrosis (Additional file 7: Figure S4, Additional file 8: Table S4).

### Identification of differentially expressed lncRNAs

**Fig. 5:**
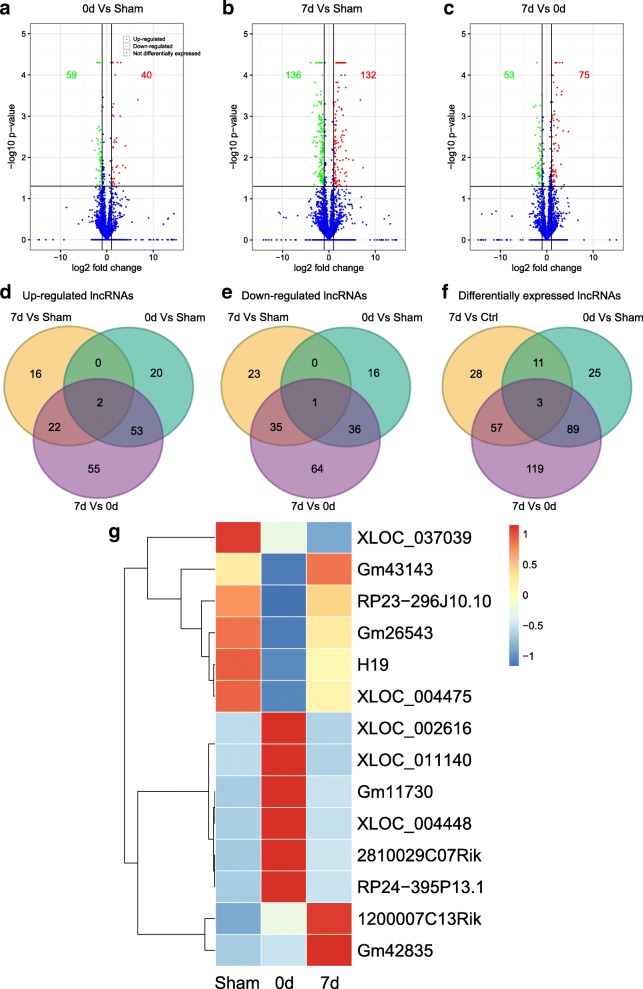
The differentially expressed (DE) profiling of long non-coding RNAs (lncRNAs). **a**–**c** Volcano plot indicating the comparisons between each pair. Only the statistically significant genes are represented in the graph. In all cases, spots colored in red and green represent upregulated and downregulated transcripts, respectively; blue represents non-differentially expressed genes. The FDR ≤ 0.01 and FC ≥ 2 were set as the thresholds to determine the significance of DETs. **d**–**f** Venn diagram showing the overlapping DETs in comparisons of each pair. Venn diagram analysis was conducted with an online tool (http://bioinformatics.psb.ugent.be/webtools/Venn/). **g** Heat map for hierarchical clustering of all of the involved DE lncRNAs

LncRNAs were screened from spliced transcripts according to the following criteria: several exons more than or equal to 2, length greater than 200 nucleotides (nt), and a FPKM value close to or higher than 0.5; and to remove overlapping genes and coding potential transcripts with database annotations at the exon region [32]. Finally, a total of 332 DE lncRNAs were identified from all of the samples (Additional file 9: Table S5). The Volcano plot and Venn diagram (Fig. 5a–f) were also used to illustrate more detailed numerical information about DE lncRNAs. The results showed that in day 0 lungs, 40 lncRNAs were upregulated and 59 were downregulated, while in day 7 lungs, 132 lncRNAs were upregulated and 136 were downregulated. Additionally, 75 lncRNAs were increased and 53 were decreased in the fibrotic lungs compared to early damaged lungs. Of special interest, 14 commonly shared DE lncRNAs were identified in the comparisons of each pair, of which one-third of the genes have never been reported in fibrogenesis (Fig. 5g).

### Functional and pathway prediction of DE lncRNAs

**Fig. 6:**
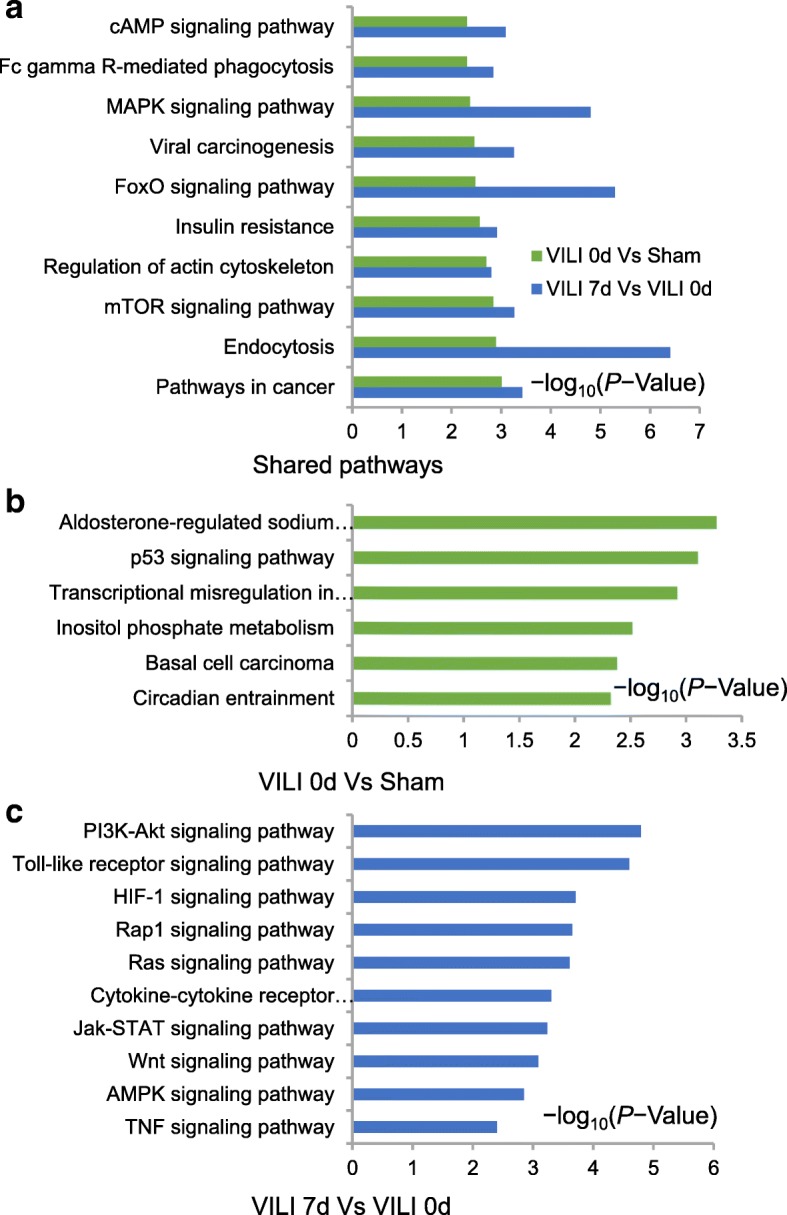
Pathway analysis of the target genes. **a** Histogram showing the pivotal enriched pathways in both stages of inflammation and fibrosis that were regulated by DE lncRNAs. **b** Histogram indicating the significant pathways that were regulated by DE lncRNAs when comparing the VILI and sham group on day 0. **c** Histogram indicating the significant pathways that were regulated by DE lncRNAs in the comparison between day 7 and day 0 in the VILI group. All analyses were performed with KOBAS 3.0, and the statistical significance was assessed using the hypergeometric test or Fisher’s exact test with the FDR correction method

In this study, 630 target genes were predicted for the 194 DE lncRNAs, and the candidate genes were further enriched into important processes and pathways with GO and KEGG analysis. Compared with sham-operated lungs, target genes of VILI on day 0 were widely distributed in the binding and intracellular regions, and were highly enriched in the processes of biological and cellular regulation and single-organism processes. This was compared with VILI lungs on day 7, in which target genes were also widely distributed in the binding, cell part, and organelle regions, and were highly enriched in the cellular process, single-organism process, and biological regulation (Additional file 10: Figure S5, Additional file 11: Table S6). KEGG analysis was performed based on the target genes. This analysis determined that signaling pathways including mTOR, FOXO, MAPK, and cAMP were implicated in lung pathophysiology, whereas PI3K/Akt, TLR, HIF-1, JAK/STAT, wingless/integrase-1 (Wnt), Ras, Rap1, and TGF-β signaling pathways appeared to play more important roles in fibrogenesis (Fig. 6, Additional file 12: Figure S6, Additional file 13: Table S7).

### Regulatory network of lncRNAs and mRNAs

**Fig. 7:**
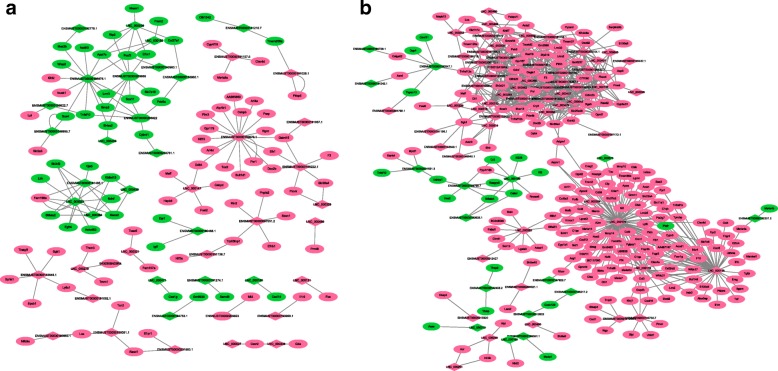
LncRNA-mRNA regulatory network analysis. **a** lncRNA-mRNA regulatory network of the VILI group compared to the sham group on day 0. **b** lncRNA-mRNA regulatory network of the VILI group on day 7 versus day 0. In all of the cases, red and green colors represent upregulated and downregulated target genes, respectively; grey solid lines indicate the prediction correlation of lncRNAs and protein coding genes resulting from the co-expression analysis and co-location analyses

To thoroughly explore the molecular mechanisms that involve lncRNAs, the lncRNA-mRNA network was constructed (Fig. 7 and Additional file 14: Figure S7). The results showed that there were a total of 152 co-expression edges connecting 143 nodes within the network of the VILI group compared to the sham group on day 0, and 738 co-expression edges connecting 292 nodes in the VILI groups on day 7 and day 0, respectively. These data may serve as a rich resource for lncRNA prediction, especially for novel genes. For example, Fig. 7a clearly shows that annotated *ENSMUST00000198222.1*, which correlated with the genes *Galnt15, Sik1, Doc2b, Pmvk, Slc38a4*, and *F3*, may participate in the stress response, stimulus response, chemical response, functional pathways of cytokine-cytokine receptor interaction, phagosome, PI3K/AKT signaling, or TNF signaling. *LNC_000147,* a novel gene that is linked to genes such as *MafF, Hspb8, Fosl2, Cebpd*, and *Ddit4* may play a similar role in cellular energy, cellular stress responses, and fibroblast proliferation during lung pathophysiology.

### qPCR validation

**Fig. 8:**
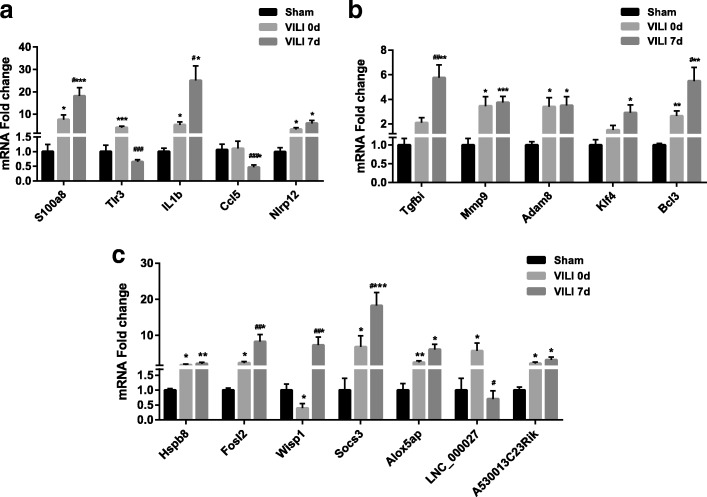
qPCR validation. **a**–**c** The relative expression levels randomly validated DETs and lncRNAs. The relative expression levels were normalized to 18S. Comparisons between two groups were determined by the Student’s *t*-test, significance level was set at: *P < 0.05, **P < 0.01, ***P < 0.001 versus sham group; ^#^*P* < 0.05, ^##^*P* < 0.01, ^###^*P* < 0.001 versus VILI group on day 0

To test the reliability of the sequence results, we randomly validated some differentially expressed genes by using qPCR, most of which have been reported as having regulatory roles in inflammation and fibrosis. For example, *S100a8* is a calcium- and zinc-binding protein that is significantly involved in the recruitment and migration of leukocytes, and cytokine and chemokine production. In addition, it functions as an alarmin or a danger associated molecular pattern molecule during the inflammatory cascade. Similarly, *Tlr3* plays a fundamental role in pathogen associated molecular pattern activation, while *IL-1b*, *Ccl5*, *Nlrp12* are implicated in the activation, chemotaxis, proliferation, differentiation, and apoptosis of immune cells. As shown in Fig. 8a, our sequencing data were in accordance with the expression patterns of these genes, suggesting that the inflammatory process represented by pathogen recognition and immunity activation play prominent roles in the early stage of VILI. Figure 8b shows the marked fibroproliferation, extracellular matrix synthesis, deposition and tight junctions in the subsequent phase on day 7. Here, *Tgfbi* was an important gene that was significantly implicated in the cell-adhesion and cell-collagen interactions. *Mmp9* and *Adam8* significantly functioned in embryonic development, reproduction, and tissue remodeling; more specifically, it was closely associated with extracellular matrix interactions. *Klf4* and *Bcl3* are the novel fibrosis-related TFs that contribute to cell proliferation and differentiation. Moreover, we chose several genes from the lncRNA-mRNA network and commonly shared DETs including *Hspb8*, *Fosl2*, *Wisp1, Socs3, Alox5ap, LNC_000027*, and *A530013C23Rik*. The verification results of these genes were consistent with our bioinformatics analysis (Fig. 8c).

## Discussion

Currently, “one-hit” and “two-hit” animal models are commonly used to generate a mechanical-based lung injury [33, 34]. Nevertheless, there are differing opinions on the fibrosis potentiation following ventilation. Cabrerabenítez et al. [12] demonstrated that high lung stretch with or without hydrochloric acid instillation could result in pulmonary fibrosis on the 8th day, and peaked on 15 days after ventilation. As expected, the “two-hit” model resulted in a greater fibroproliferative response than the “one-hit” model. In contrast, Curley et al. [13] found that VILI generated marked but transient fibrotic alternations within 24 h, and the anatomical lung structure was restored after 1–2 weeks. This experiment was also supported by Villar et al. [14], who proposed that lung fibrosis occurred immediately after 4 h high stretch ventilation in a mouse model of sepsis-induced acute lung injury. To address this issue, we monitored our “one-hit” mice for up to 1 month. Pathological staining and measurement of hydroxyproline content and TGF-β1 indicated an overall alteration from early inflammation to fibrogensis. It is noteworthy that fibrosis signature caused by MV was most pronounced on day 7 and was not the same as fibrosis induced by bleomycin [35]. The fibrosis induced by MV was more likely to manifest as local lesions around the lung interstitium, airway and vessels, which recovered within 1 month.

Based on a well-defined animal model, we performed whole transcriptome analysis to determine the potential molecular mechanisms in lung pathophysiology post-ventilation. Previously, transcriptomics was performed to explore the potential mechanisms of VILI, or protective ventilation use against VILI at a genetic level [36, 37]. Different from the previous analyses, the current study did not solely focus on studying dysregulated genes or pathways involved in the early stage of VILI. Rather, this was the first transcriptomics study to reveal a broad spectrum of dysregulated transcripts and potential molecular pathways involved in the VILI fibrotic process.

From pairwise comparisons among the sham and VILI groups on day 0 and day 7, a total of 1297 dysregulated DETs were identified after ventilation. We mainly analyzed the expression patterns of the 63 commonly shared DETs, which allowed for a better understanding of the probable biological and regulatory functions of the involved transcripts. For example, the *Hspa1a, Il7r, Sult1a1, Abcg2*, and *Stc1* genes exhibited increased activity at the acute injury phase, and returned to baseline levels after 7 days. The expression levels of *Wisp1, Clca1*, and *Hic1* showed a rapid initial decline and then increased after 7 days, suggesting their critical role in inflammation, the innate immune response, and tissue repair and resolution. Subsequently, 79 TFs were profiled among the total DTEs, and they showed extensive regulatory functions in organ development, immune response, and homeostasis maintenance; and in lung injury, repair and regeneration. In this study, three commonly shared TFs were detected with statistical significance either in the early inflammation or subsequent phase of fibrosis. These included *MafF*, a basic region leucine zipper-type TF important for cellular stress regulation [38] that was increased 3.36-fold in mice subjected to ventilation and increased 2.17-fold after 7 days. *Hic1*, a gene annotated as hypermethylated in cancer 1 that was decreased 2.55-fold in lungs at weaning and returned to baseline levels at increased with 2.33-fold 7 days later. This gene was formerly considered to be a strong candidate as a tumor suppressor gene because it is a master modulator of cell apoptosis and DNA damage survival [39]. *Fosl2*, an important gene in the Fos family, was increased 2.99-fold in the VILI group on day 0 compared to the controls, and increased 2.07-fold in the VILI group on day 7 versus day 0. Studies have shown that *Fosl2* is a novel mediator of cell proliferation, differentiation, and transformation in the fibrotic pathophysiology of certain diseases, such as scleroderma and pulmonary hypertension [40]. The identification of *Fosl2* was not unexpected; however, the two other genes (*MafF* and *Hic1*) were not previously found in mouse models of lung injury, fibrosis, or in ventilation research. Additionally, other factors identified in the VILI group versus sham group on day 7, such as *Hes2, Ikzf3*, and *Mafb*, have not been previously reported in similar diseases or models. These TFs may be potential candidate genes for predicting disease mechanisms or intervention targets.

The results also determined substantially enriched pathways that were dysregulated in the respective phases of inflammation and fibrosis. For example, mTOR signaling is a central regulator activated in response to growth factors, nutritional status, and stress signals [41]. JAK/STAT signaling pivotally regulates cell growth, proliferation, differentiation, migration, and apoptosis [42]. cAMP signaling mainly functions in cell chemotaxis, immune mediator induction, and inflammatory response regulation, with a correlation to microbial and cardiovascular pathogenesis [43]. In the current study, these three pathways were all significantly implicated in the regulation of early inflammation. With an intensive focus on fibrosis-promoting pathways, the TGF-β, HIF-1, TLR, and NF-κB signaling pathways were found to be key in fibrogenesis. Accordingly, it is well recognized that overactivation of the TGF-β signaling pathway is one of the most commonly characterized events in the regulation of fibrosis. In addition, TGF-β1 is considered the most central mediator in the activation, proliferation, and differentiation of epithelial cells, myofibroblasts, excessive production of extracellular matrix (ECM), and inhibition of ECM degradation. Apart from canonical (SMAD-based) signaling pathways, non-canonical (non-SMAD-based) pathways such as MAPK, p53, and Notch signaling have also been explored in bleomycin-induced lung fibrosis as well as fibrosis in other organs [44]. HIF-1 and TLR signaling function as master regulatory signaling pathways in cellular and systemic homeostatic responses to hypoxia as well as natural and acquired immune responses to pathogens, both of which have been increasingly implicated in pulmonary fibrosis [45]. However, these pathways have not been associated with the pathogenesis of VILI fibrosis.

Importantly, the results of this study contribute to a consistent emerging picture of critical non-coding transcripts involved in lung pathophysiology. LncRNAs are an abundant class of transcripts with no coding function and typical lengths of > 200 nt [46–48]. Over the last decade, many lung diseases have been associated with dysregulated lncRNAs, such as idiopathic pulmonary fibrosis, chronic obstructive pulmonary disease or pulmonary hypertension [49, 50]. However, to the best of our knowledge, no studies have focused on dysregulated or dysfunctional lncRNAs that may be responsible for the pathogenesis of VILI or subsequent fibrosis. Thus, there is a need to identify functional non-coding transcripts from a vast transcriptome, to provide a better perspective of the molecular mechanisms and therapeutic targets of the disease.

In total, this study profiled 332 differentially altered lncRNAs and well-established lncRNA-mRNA networks in the comparisons between each pair, nearly a quarter of which were novel genes without annotations. Here, 14 common DE lncRNAs that may serve as signals, decoys, guides, or scaffolds for regulating gene expression in lung injury or fibrosis are listed. For example, *1200007C13Rik*, a gene that reportedly plays a master function in organ development and repair, may also be an important candidate lncRNA with potential implications in fibrogenesis following ventilation. Other genes, such as *LNC_000027*, which is located on chr4 from chr1:74175576–74,180,761 with a predicted target gene of *Cxcr2*, is considered to be a master mediator for neutrophil migration and activation during the inflammatory response [51]. Through target prediction and enrichment analysis, we found that the identified DE lncRNAs likely participate in the processes of cellular and biological regulation through the mTOR, FOXO, MAPK, and cAMP signaling pathways. We speculated that DE lncRNA likely regulates fibrosis through signaling pathways such as PI3K/Akt, TLR, HIF-1, and Wnt signaling. Wnt signaling is a classical developmental pathway required for proper organ development. The overactivation of both canonical and non-canonical signaling has been identified and established in a variety of fibrotic diseases [52]. Previously, Villar and his co-workers [18, 53] showed that the Wnt/β-catenin signaling pathway is modulated very early by MV in VILI, and in lung repair without pre-existing lung disease. This suggests an attractive candidate for the prevention and/or management of VILI. However, the majority of identified pathways have not been considered in MV-associate lung fibrosis, and no studies have associated these pathways with the regulatory role of lncRNAs. Therefore, these observations may provide a novel perspective into potential molecular mechanisms for further research.

This study had several limitations. First, our “one-hit” mouse model did not fully represent the real-life “two-hit” conditions seen in humans. It should also be recognized that even if a mouse model used for VILI or fibrosis is validated, it is not fully representative of the classical recommended parameters of ventilation seen in humans. Therefore, bias in comparison to what occurs in humans may develop. Second, our initial results were interpreted mainly based on bioinformatics and literature analysis. It may be necessary confirm our findings using functional or mechanistic studies at the protein level. Nevertheless, the results of this study provide novel perspectives into the potential molecular mechanisms underlying VILI and subsequent fibrosis, providing a foundation for future research studies.

## Conclusions

This is the first transcriptomic study to reveal all of the transcript expression patterns and critical pathways involved in MV-associated lung fibrosis, as well as the first preliminary study to identify the important DE lncRNAs that regulate inflammation and fibrosis. We speculated that TGF-β, HIF-1, TLR, and NF-κB signaling may be the most important pathways that participate in fibrogensis, and that DE lncRNAs may regulate the fibrotic response through Wnt, HIF-1, and TLR signaling pathways. These data provide novel perspectives into potential molecular mechanisms for further research.

## Additional files

Additional file 1: **Figure S1.** Primers designed for qPCR validation. (PDF 166 kb)

Additional file 2: **Table S1.** The entire list of differentially expressed transcripts (DETs) in the pairwise comparisons among the sham and VILI groups on day 0 and day 7. (XLSX 266 kb)

Additional file 3: **Figure S2.** The profiling of DE mRNAs in Volcano plot and Venn diagram. (PDF 8628 kb)

Additional file 4: **Table S2.** The entire list of dysregulated transcription factors (TFs) in the comparisons of each pair. (XLSX 62 kb)

Additional file 5: **Figure S3.** The summary histogram of the top 20 dysregulated GO terms of DE mRNAs in the comparisons of each pair. (PDF 211 kb)

Additional file 6: **Table S3.** The entire list of significant dysregulated GO terms of DE mRNAs in the comparisons of each pair. (XLSX 1870 kb)

Additional file 7: **Figure S4.** The summary scatterplot of the top 20 dysregulated pathways of DE mRNAs in the comparisons of each pair. (PDF 239 kb)

Additional file 8: **Table S4.** The entire list of dysregulated functional pathways of DE mRNAs in the comparisons of each pair. (XLSX 94 kb)

Additional file 9: **Table S5.** The entire list of DE lncRNAs in the comparisons of each pair. (XLSX 100 kb)

Additional file 10: **Figure S5.** The summary histogram of the top 20 dysregulated GO terms of terget genes in the comparisons of each pair. (PDF 198 kb)

Additional file 11: **Table S6.** The entire list of significant dysregulated GO terms of target genes in the comparisons of each pair. (XLSX 2312 kb)

Additional file 12: **Figure S6.** The summary scatterplot of the top 20 dysregulated pathways of target genes in the comparisons of each pair. (PDF 238 kb)

Additional file 13: **Table S7.** The entire list of dysregulated functional pathwyas of target genes in the comparisons of each pair. (XLSX 66 kb)

Additional file 14: **Figure S7.** LncRNA-mRNA regulatory network of the VILI group compared to the sham group on day 7. (PDF 547 kb)

## Abbreviations

ARDS: Acute respiratory distress syndrome
BALF: Bronchoalveolar lavage fluid
cAMP: Cyclic adenosine monophosphate
DETs: Differentially expressed transcripts
ECM: Extracellular matrix
EEP: Positive end-expiratory pressure
FDR: False discovery rate
FOXO: Forkhead box protein O
FPKM: Fragments per kilo-base of exon per million fragments mapped
GO: Gene Ontology
HIF-1: Hypoxia inducible factor-1
JAK/STAT: Janus kinase-signal transducer and activator of transcription
KEGG: Kyoko Encyclopedia of Genes and Genomes
KOBAS: KO-Based Annotation System
LncRNAs: Long non-coding RNAs
MAPK: Mitogen-activated protein kinase
mTOR: Mechanistic target of rapamycin
MV: mechanical ventilation
NF-κB: Factor kappa-light-chain-enhancer of activated B cells
PI3K/Akt: Phosphoinositide 3-kinase/Akt
TF: Transcription factor
TGF-β1: Transforming growth factor beta 1
TLR: Toll-like receptor
TNF: Tumor necrosis factor
VILI: Ventilator-induced lung injury
VT: Tidal volume
Wnt: Wingless/integrase-1
α-SMA: alpha smooth muscle actin
