# Supplementary material for: Landscape of transcription and long non-coding RNAs reveals new insights into the inflammatory and fibrotic response following ventilator-induced lung injury
Source: Respir Res. 2018 Jun 22;19:122. doi: 10.1186/s12931-018-0822-z (PMC6013938; doi:10.1186/s12931-018-0822-z)
Supplement: Supplementary file 1 — Figure S1. Primers designed for qPCR validation. (PDF 166 kb) [file 12931_2018_822_MOESM1_ESM.pdf]

Gene symbol	Forward primer	Reverse primer
S100a8	5’-CCTTGAGCAACCTCATTGATGTC-3’	5’-CCTCGAAGTTAATTGCATTGTCAC-3’
Tlr3	5’-GGTTGACGCACCTGTTCTCTATC-3’	5’-CTGTATCATATTCTACTCCTTGC-3’
IL-1β	5’-TTCCTTGTGCAAGTGTCTGAAGC-3’	5’- GAAGCTGGATGCTCTCATCAGG-3’
Ccl5	5’-CACGTCAAGGAGTATTTCTACACC-3’	5’-CTTGAACCCACTTCTTCTCTGG-3’
Nlrp12	5’-ACCTGCAGACTCCAGAAGCTGT-3’	5’-CACAGCAGACAGACACCTGTGTC-3’
Tgfbi	5’-CCAGATCCATCACTATCCCAATG-3’	5’-CTGGATGTTGTTGGTGATGGTG-3’
Mmp9	5’-ACTTTTGTGGTCTTCCCCAAAG-3’	5’-GAGACTGCTTCTCTCCCATCATC-3’
Adam8	5’-TGAGGTGACAGAGCAACTGCAG-3’	5’-TCAAGTGGACAGTGGACCCAAC-3’
Klf4	5’-GTTGACTTTGGGGCTCAGGTAC-3’	5’-GACGCAGTGTCTTCTCCCTTC-3’
Bcl3	5’-GTGGATGAGGATGGAGACACG-3’	5’-AGGTTGTTATGGACGTCTACTTCG-3’
Hspb8	5’-GAAGAGCTGATGGTAAAGACCAAG-3’	5’-CAAATACAGTGGCTGGATCCAC-3’
Fosl2	5’-CTATCCACGCTCACATCCCTAC-3’	5’-CTTCTCCTCCTCCTCAGGAGAC-3’
Wisp1	5’-GTAGCTCCTGTGACGCTGACTTC-3’	5’-TCGTTTCCTCTAGTGGTGCTGG-3’
Socs3	5’- GATTTCGCTTCGGGACTAGCTC-3’	5’-AAACTTGCTGTGGGTGACCATG-3’
Alox5Ap	5’- CTAGCACCAGCCTGGTTGTCTC-3’	5’-TTGCTTTCATGCTCCACCTTG-3’
LNC_000027	5’-GCAATCAAAGCAATTCATGGAAC-3’	5’-CTCTTCACTGCATCAGCCTTGAC-3’
A530013C23Rik	5’-TAGGAAAGGCTAGTCCAGACAGC-3’	5’-GAGAATGTAGTCTCTCCACAAGGC-3’
18S	5’- CACGGACAGGATTGACAGATTG-3’	5’- GCGTAACTAGTTAGCATGCCAGAG-3’
