# Supplementary figures and images for: Landscape of transcription and long non-coding RNAs reveals new insights into the inflammatory and fibrotic response following ventilator-induced lung injury
Source: Respir Res. 2018 Jun 22;19:122. doi: 10.1186/s12931-018-0822-z (PMC6013938; doi:10.1186/s12931-018-0822-z)

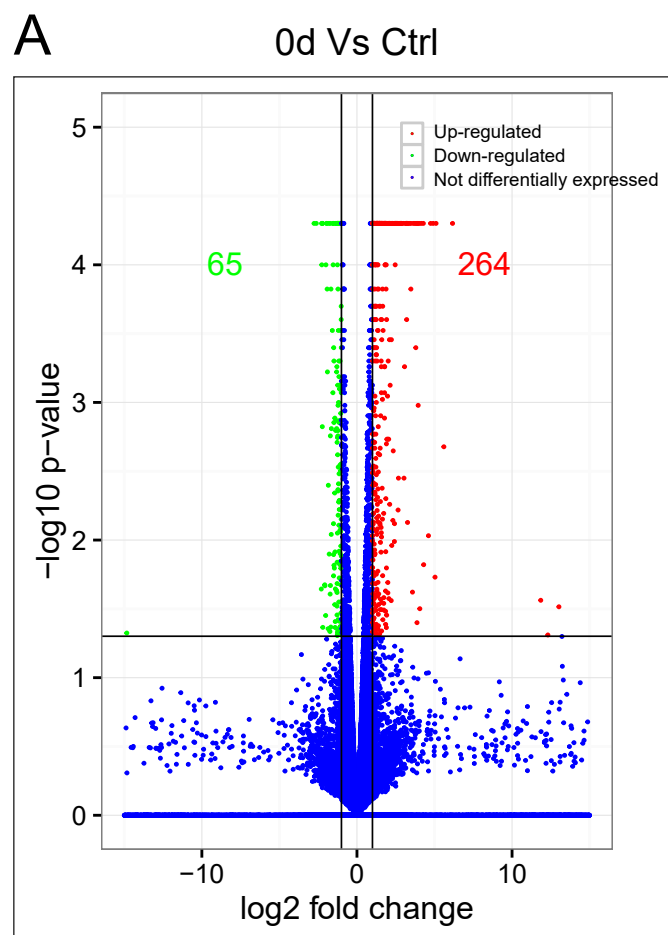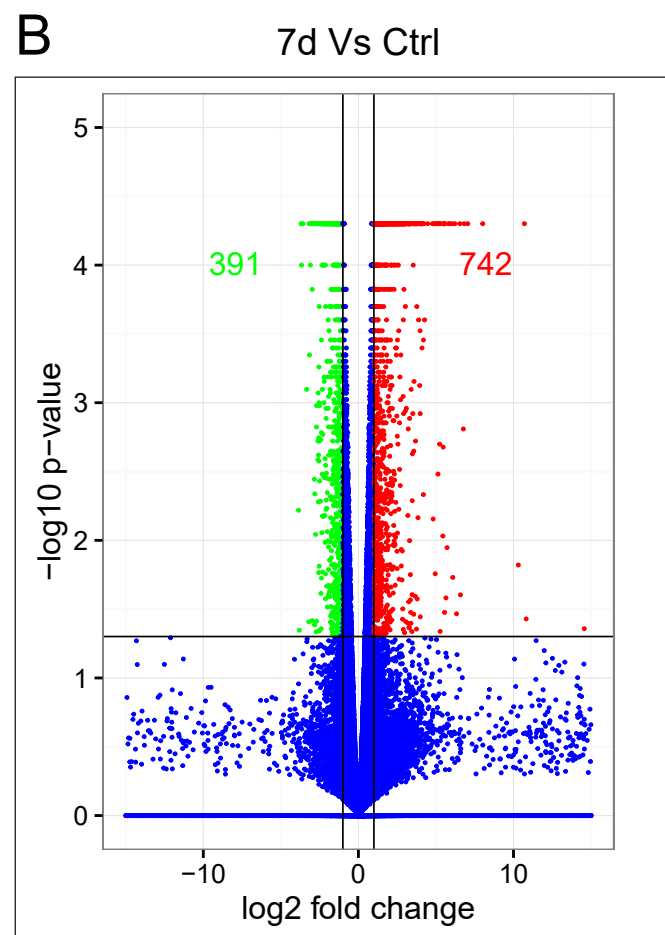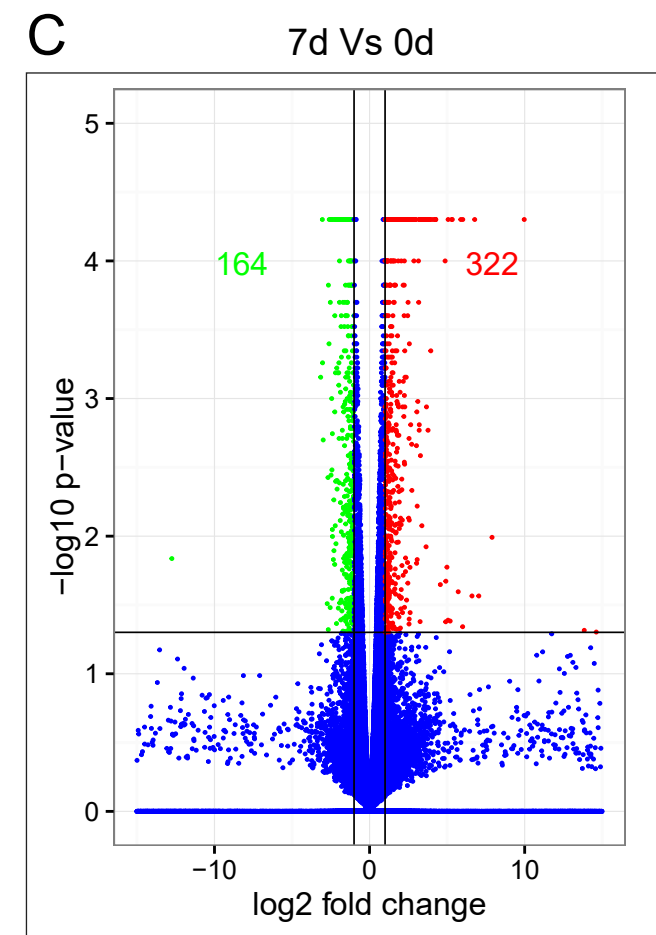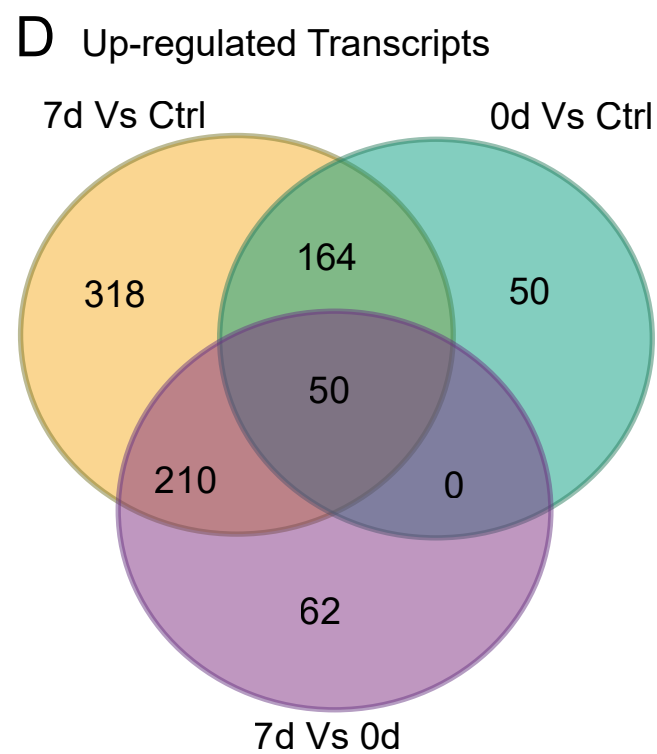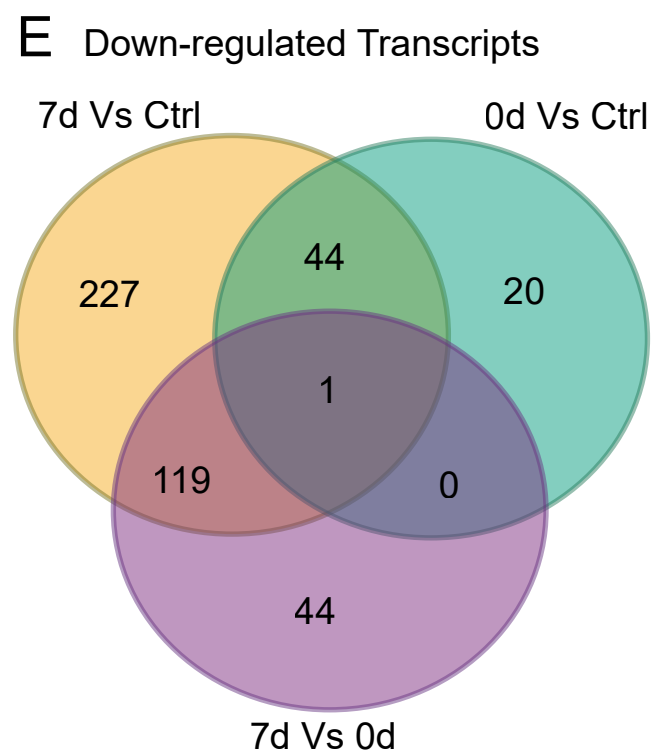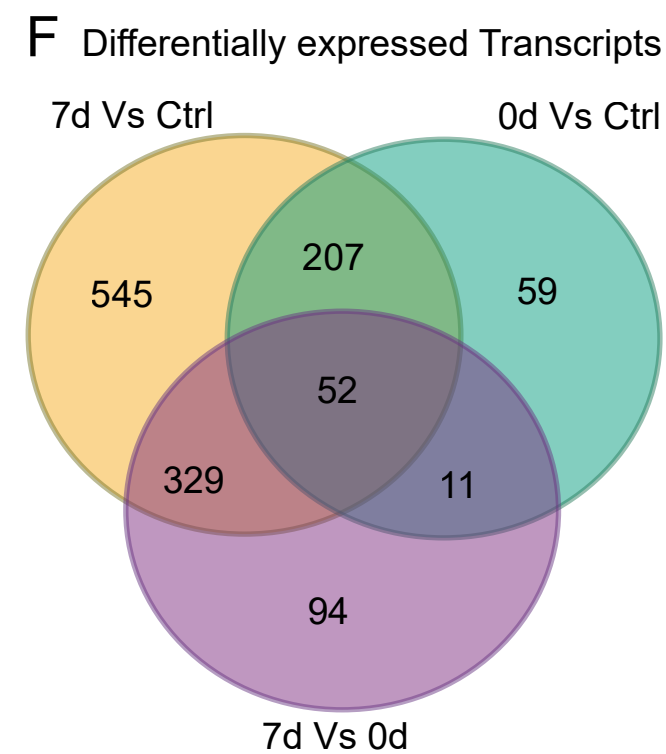

Supplement: Supplementary file 3 — Figure S2. The profiling of DE mRNAs in Volcano plot and Venn diagram. (PDF 8628 kb) [file 12931_2018_822_MOESM3_ESM.pdf]

A

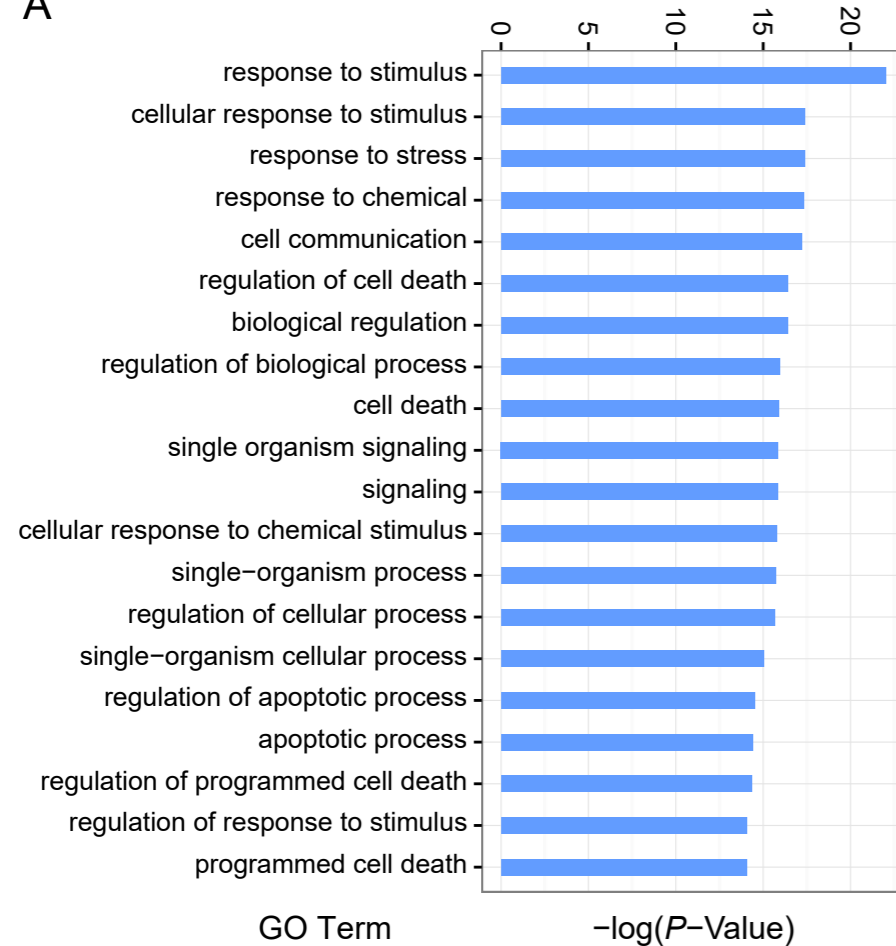

B

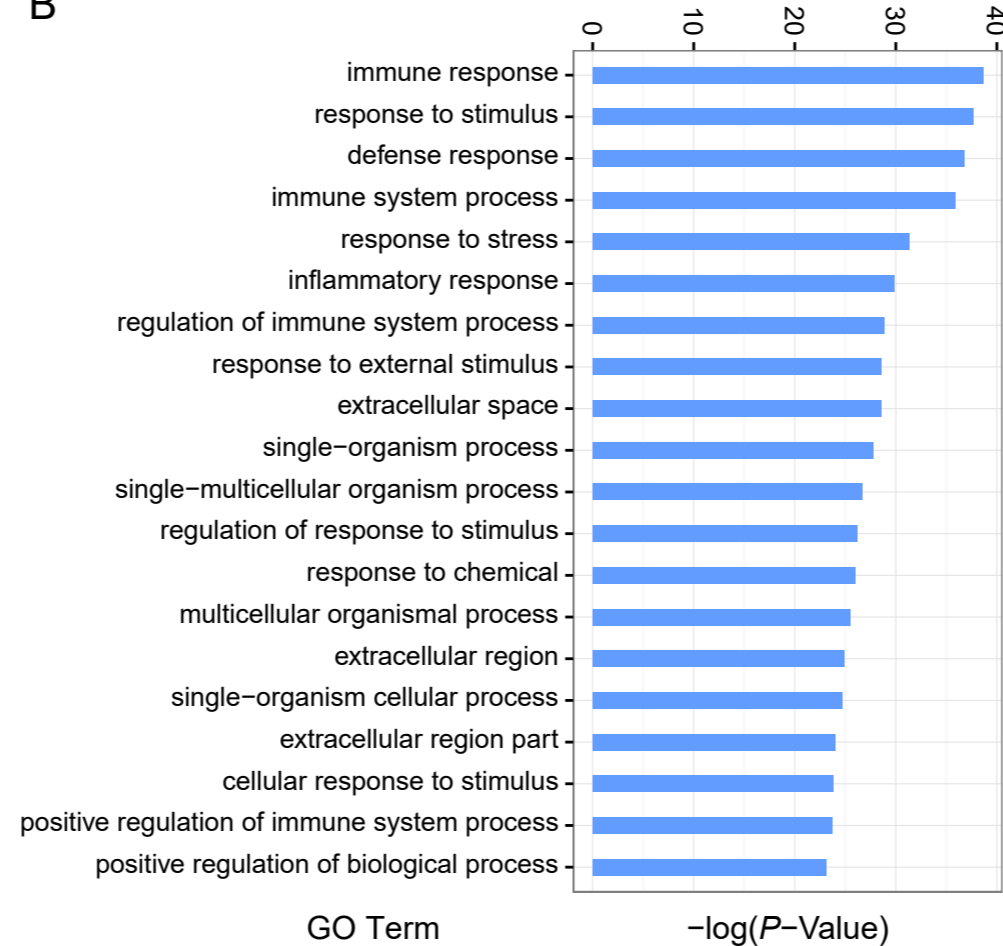

C

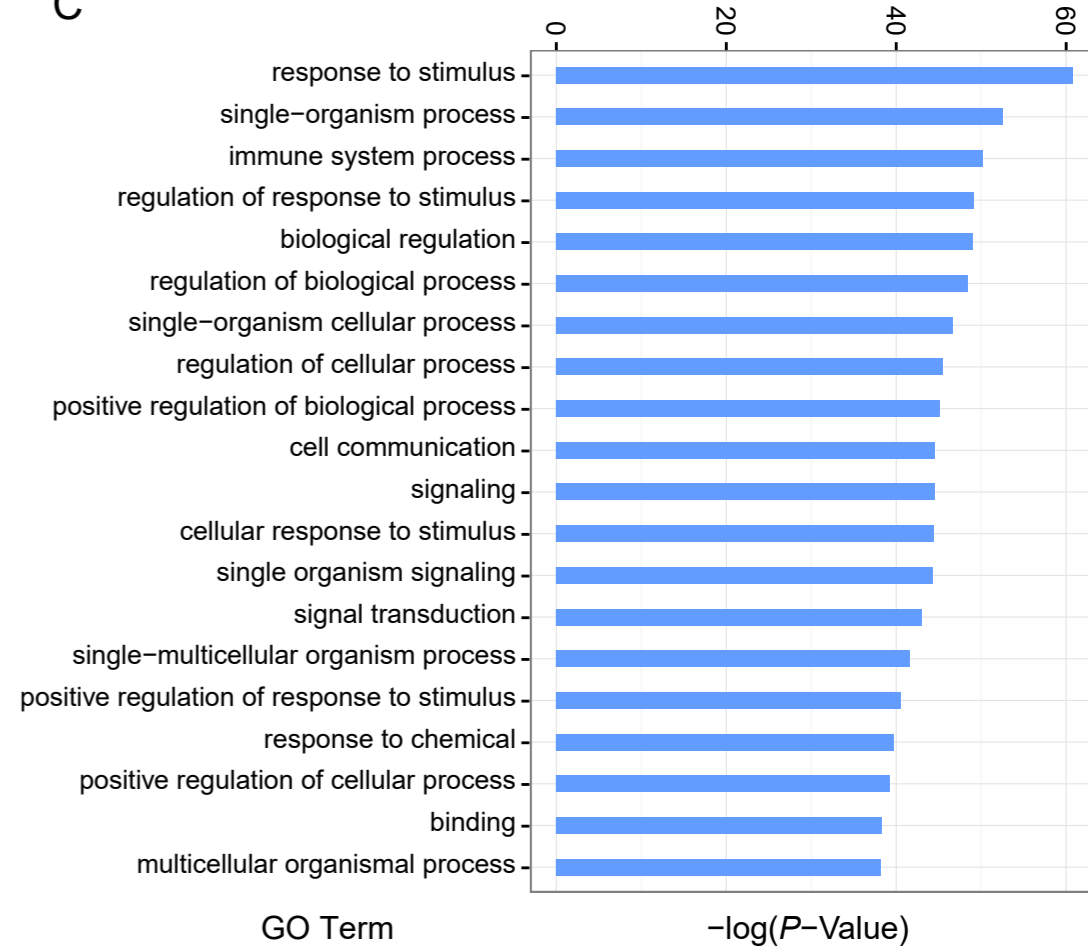

Supplement: Supplementary file 5 — Figure S3. The summary histogram of the top 20 dysregulated GO terms of DE mRNAs in the comparisons of each pair. (PDF 211 kb) [file 12931_2018_822_MOESM5_ESM.pdf]

A

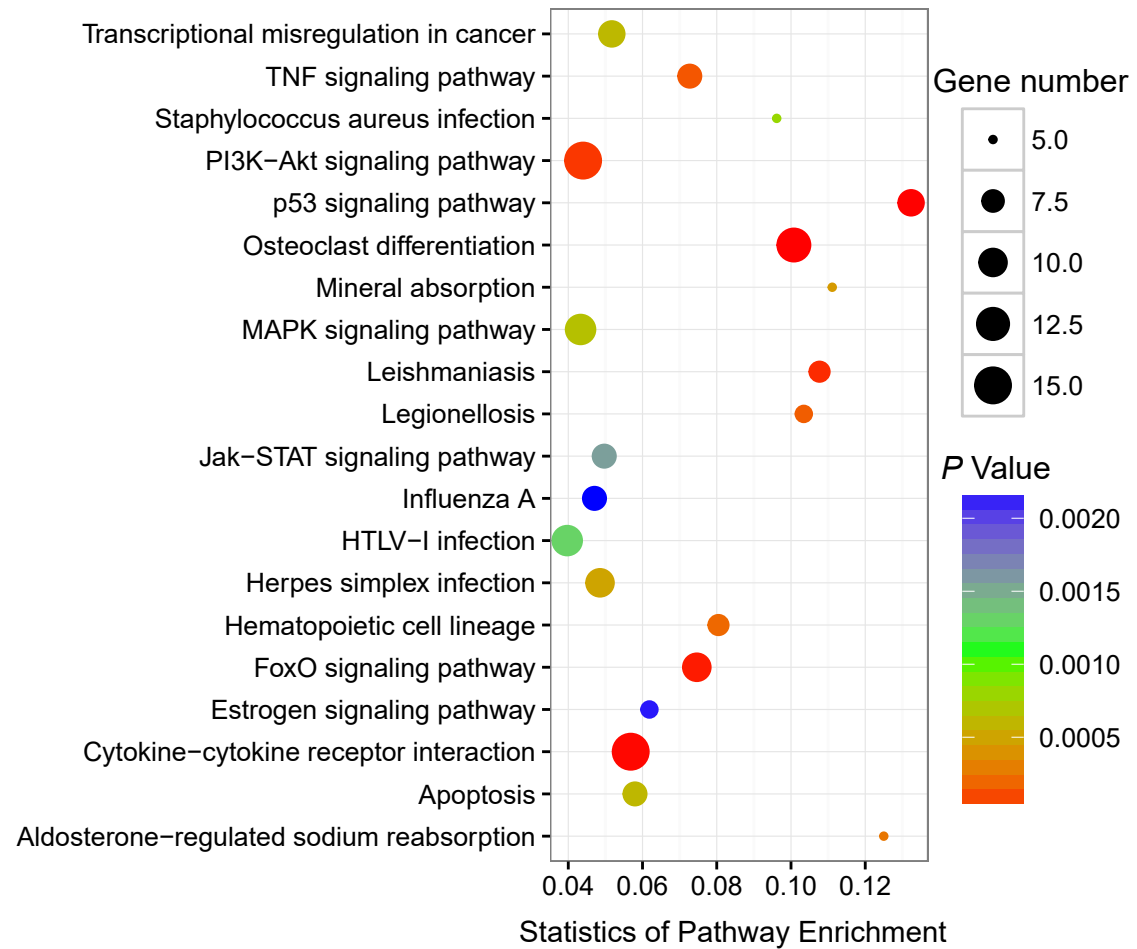

B

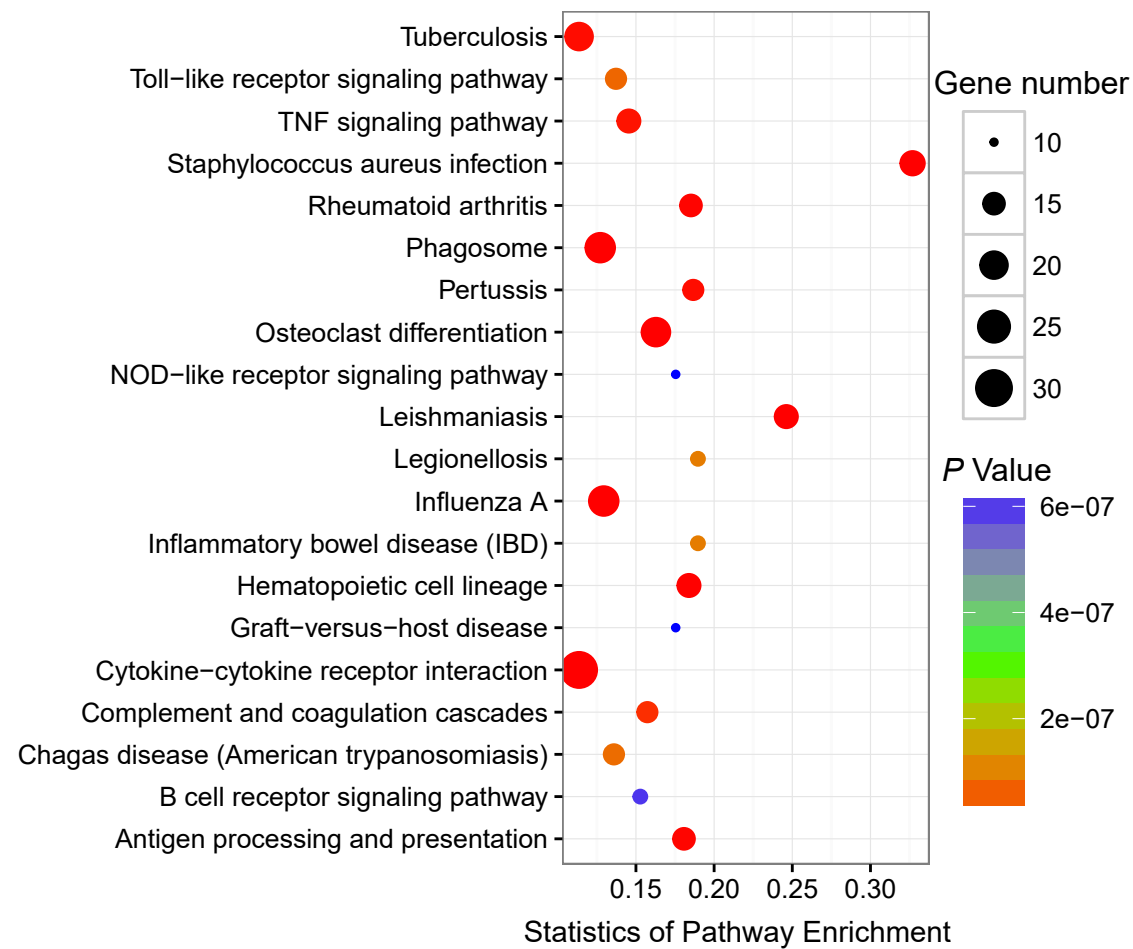

C

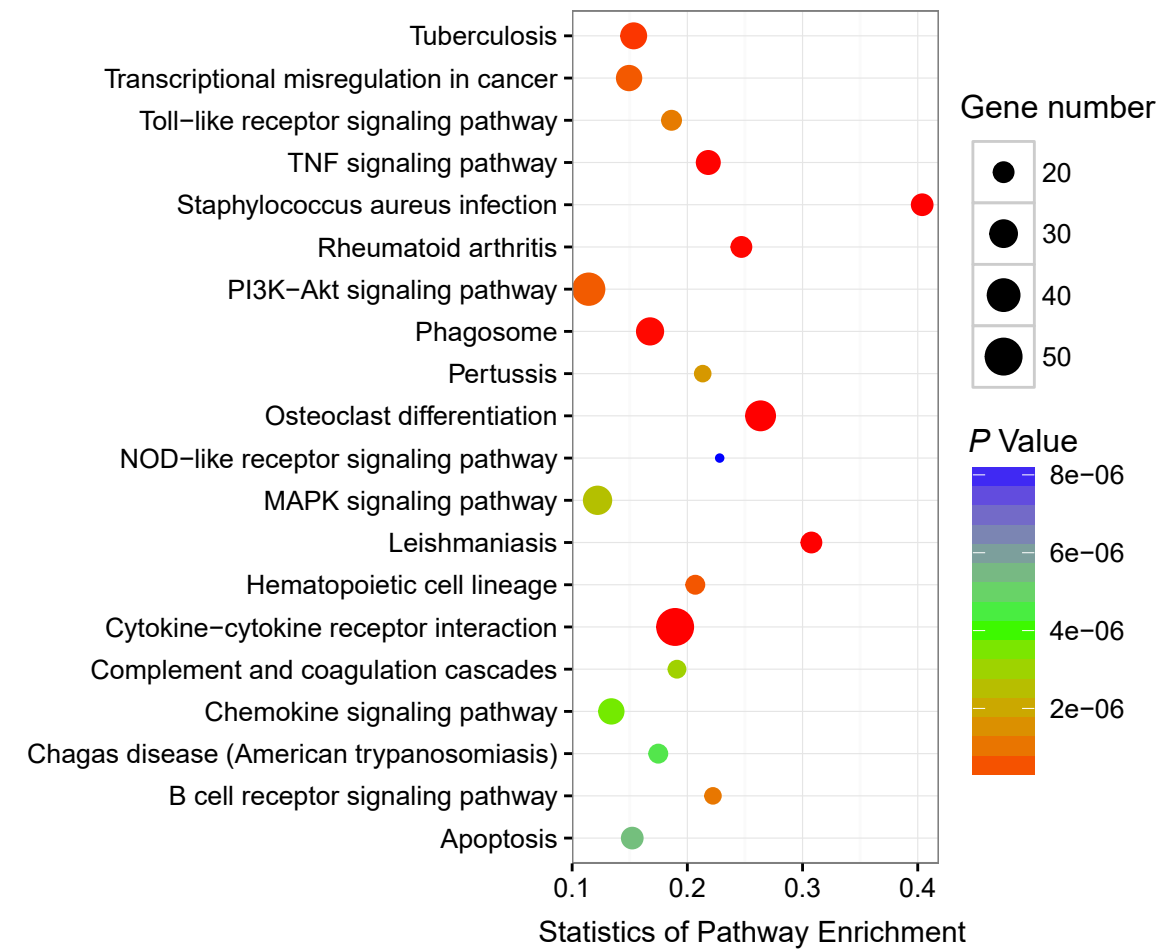

Supplement: Supplementary file 7 — Figure S4. The summary scatterplot of the top 20 dysregulated pathways of DE mRNAs in the comparisons of each pair. (PDF 239 kb) [file 12931_2018_822_MOESM7_ESM.pdf]

A

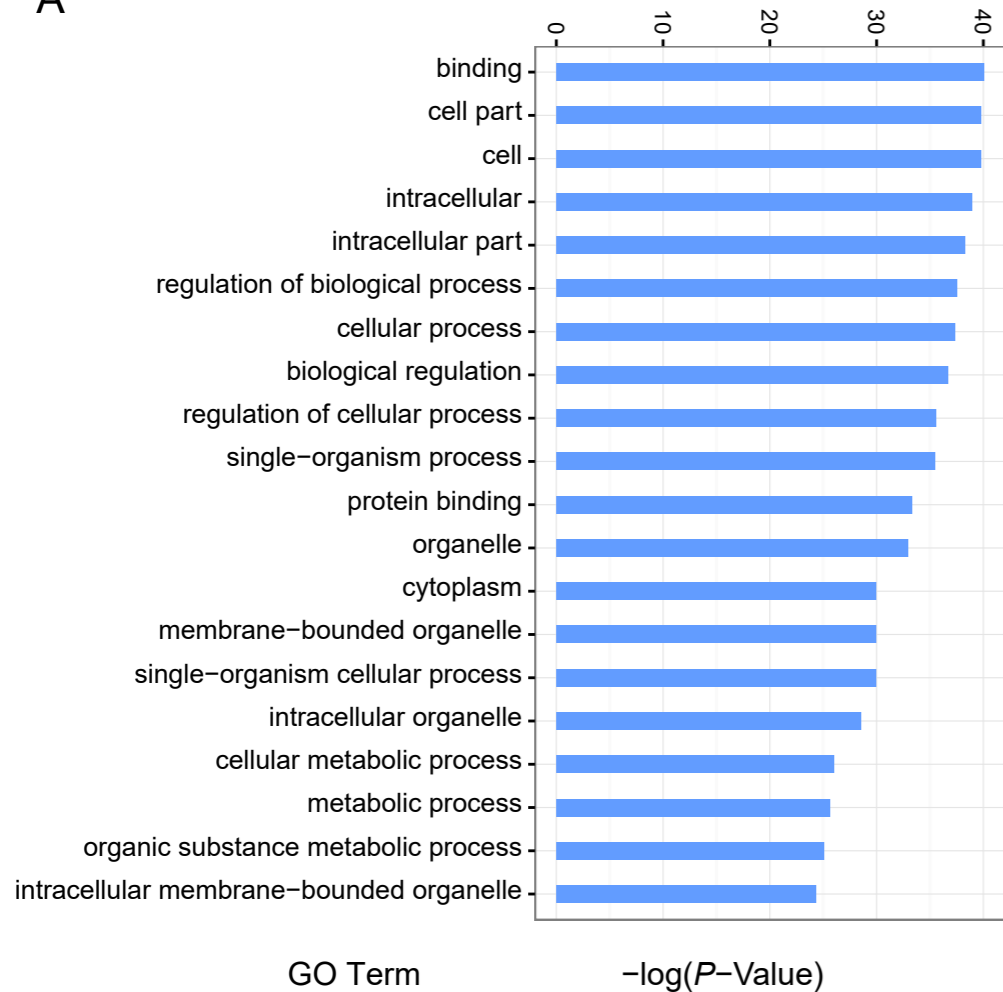

B

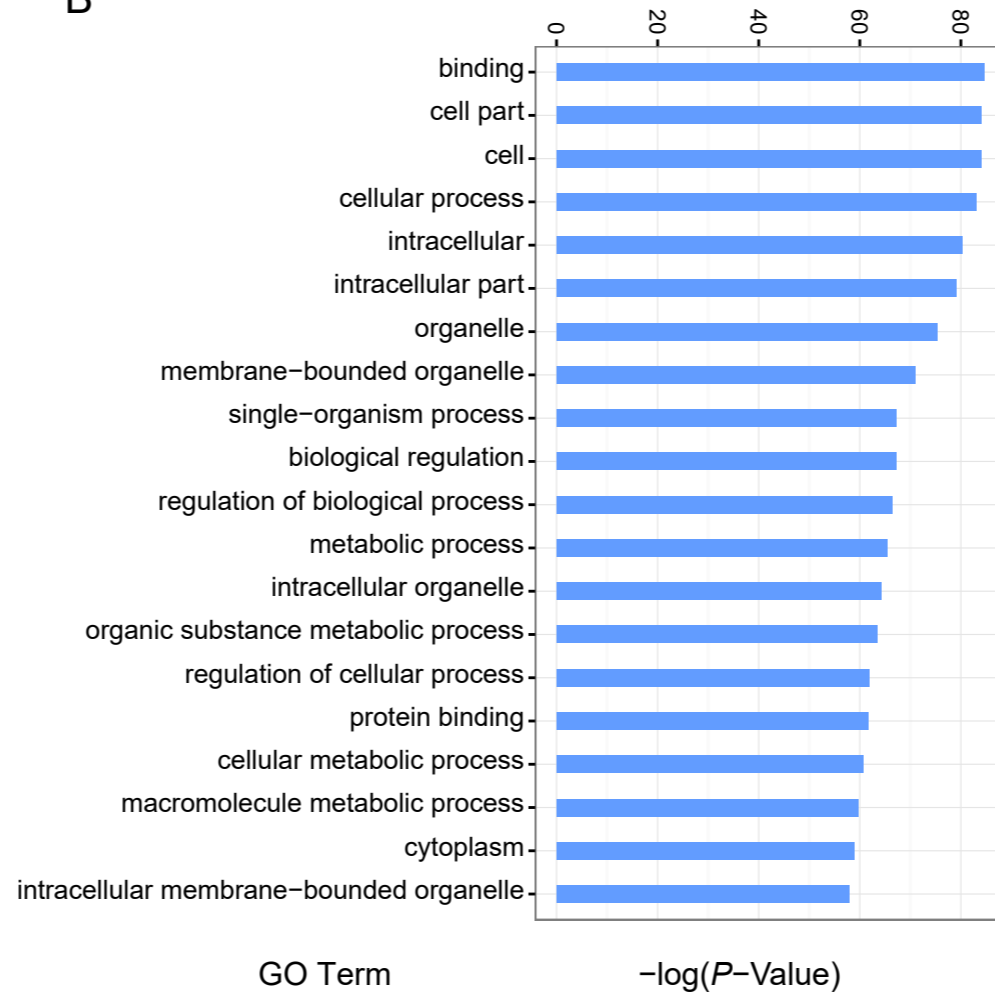

C

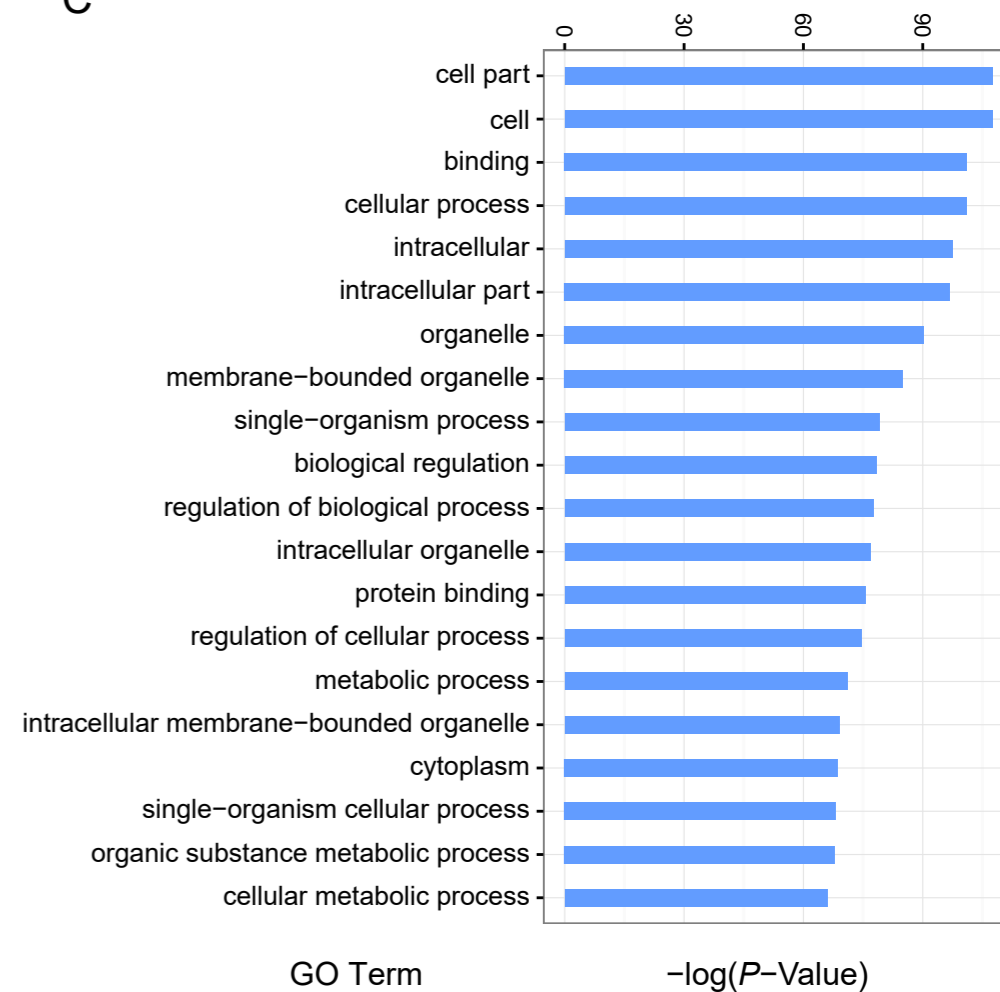

Supplement: Supplementary file 10 — Figure S5. The summary histogram of the top 20 dysregulated GO terms of terget genes in the comparisons of each pair. (PDF 198 kb) [file 12931_2018_822_MOESM10_ESM.pdf]

A

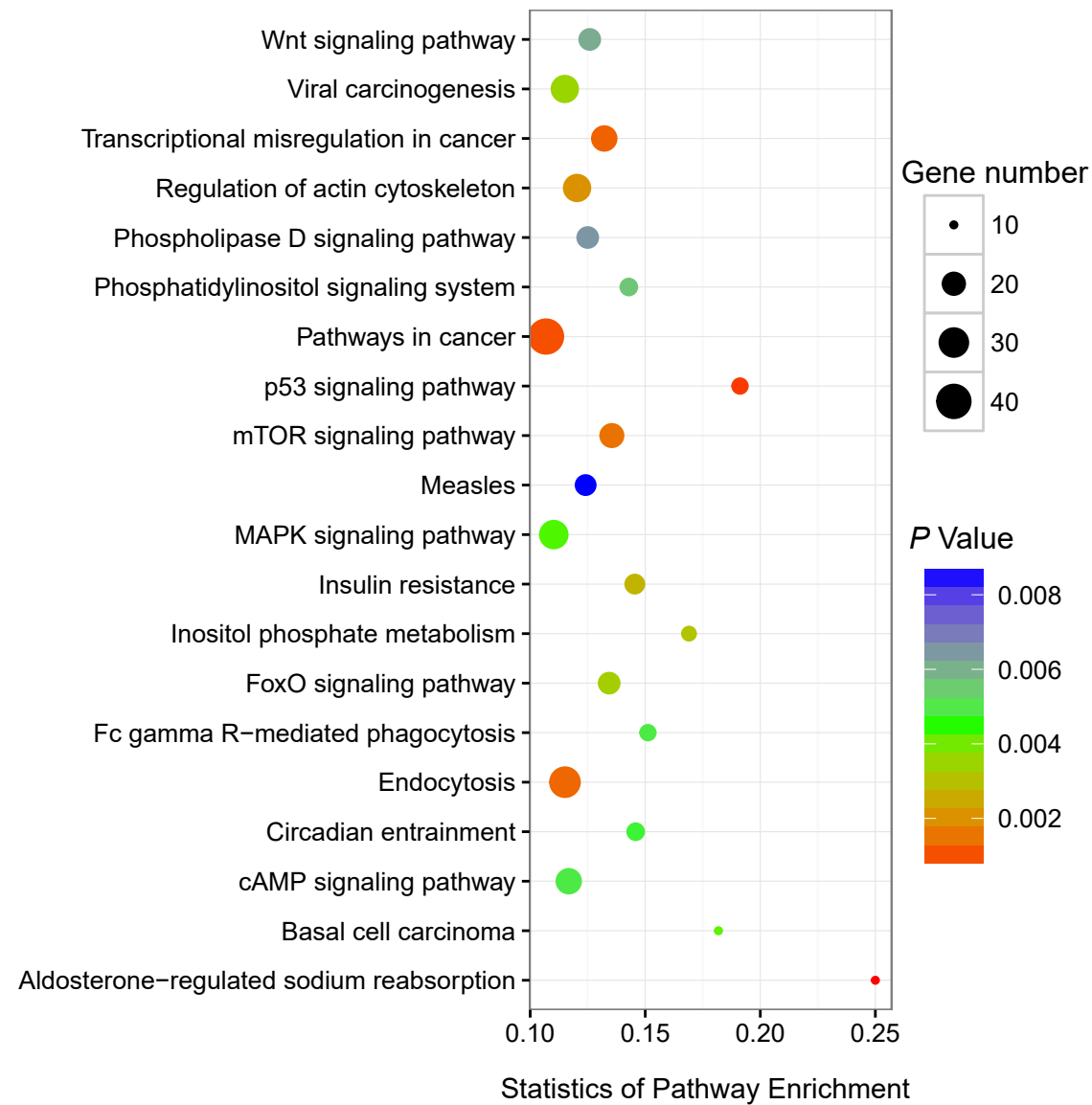

B

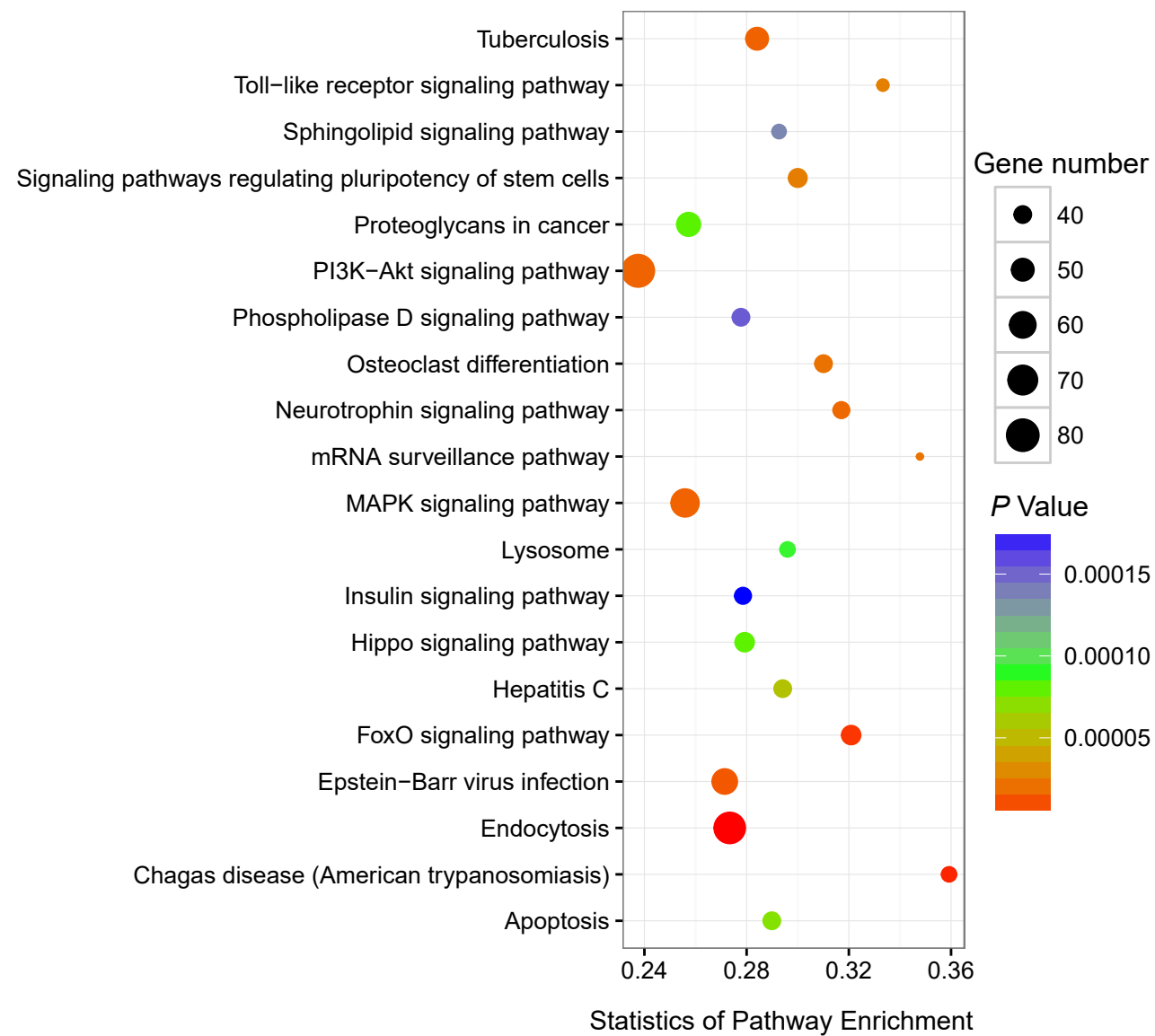

C

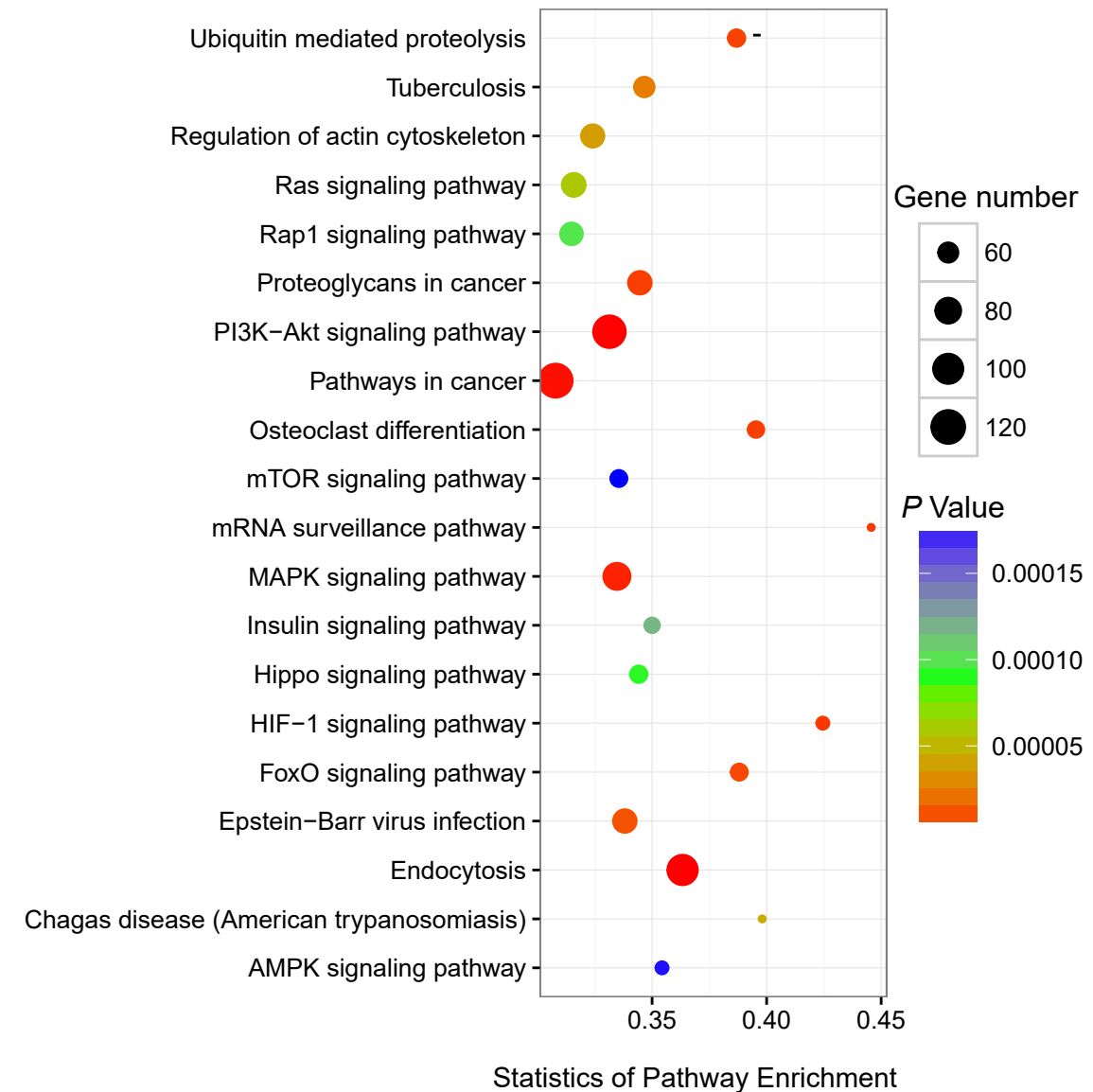

Supplement: Supplementary file 12 — Figure S6. The summary scatterplot of the top 20 dysregulated pathways of target genes in the comparisons of each pair. (PDF 238 kb) [file 12931_2018_822_MOESM12_ESM.pdf]

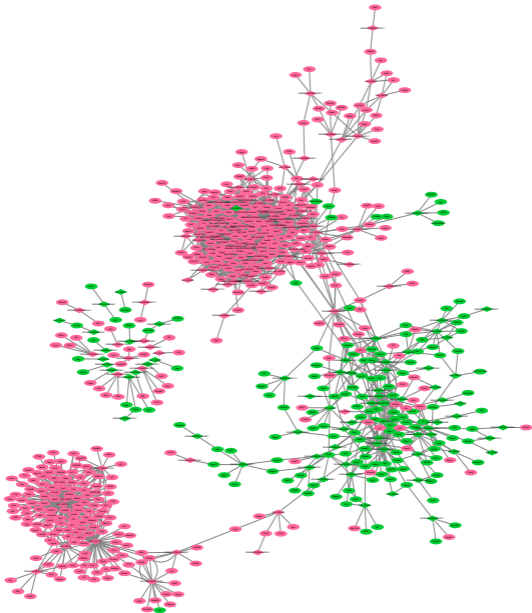

Supplement: Supplementary file 14 — Figure S7. LncRNA-mRNA regulatory network of the VILI group compared to the sham group on day 7. (PDF 547 kb) [file 12931_2018_822_MOESM14_ESM.pdf]
